# Virome of *Rhipicephalus* ticks by metagenomic analysis in Guangdong, southern China

**DOI:** 10.3389/fmicb.2022.966735

**Published:** 2022-08-11

**Authors:** Luanying Guo, Jun Ma, Junwei Lin, Meiyi Chen, Wei Liu, Jin Zha, Qinqin Jin, Hongrong Hong, Weinan Huang, Li Zhang, Ketong Zhang, Zhengkai Wei, Quan Liu

**Affiliations:** ^1^School of Life Sciences and Engineering, Foshan University, Foshan, China; ^2^Jieyang Animal Health Supervision Institute, Jieyang, China; ^3^Agricultural and Rural Bureau of Huilai County, Jieyang, China; ^4^College of Veterinary Medicine, Northeast Agricultural University, Harbin, China; ^5^Zhongkai University of Agriculture and Engineering, Guangzhou, China; ^6^Center for Infectious Diseases and Pathogen Biology, International Center of Future Science, Key Laboratory of Organ Regeneration and Transplantation of the Ministry of Education, The First Hospital, Jilin University, Changchun, China

**Keywords:** tick-borne viruses, viral diversity, *Rhipicephalus* ticks, meta-transcriptome, Guangdong

## Abstract

Tick-borne viruses (TBVs) have increasingly caused a global public health concern. This study collected *Rhipicephalus* ticks in Guangdong, southern China to identify RNA viruses. Meta-transcriptome analysis revealed the virome in *Rhipicephalus* ticks, resulting in the discovery of 10 viruses, including Lihan tick virus, Brown dog tick phlebovirus 1 and 2 in the family *Phenuiviridae,* Mivirus and Wuhan tick virus 2 in the family *Chuviridae*, Wuhan tick virus 1 in the family *Rhabdoviridae,* bovine hepacivirus in the family *Flaviviridae*, Guangdong tick quaranjavirus (GTQV) in the family *Orthomyxoviridae*, Guangdong tick orbivirus (GTOV) in the family *Reoviridae*, and Guangdong tick Manly virus (GTMV) of an unclassified family. Phylogenetic analysis showed that most of these TBVs were genetically related to the strains in countries outside China, and GTQV, GTOV, and GTMV may represent novel viral species. These findings provided evidence of the long-distance spread of these TBVs in Guangdong, southern China, suggesting the necessity and importance of TBV surveillance.

## Introduction

Ticks (Acari: Ixodoidea) are considered to be next to mosquitoes as vectors for pathogenic agents. Tick-borne viruses (TBVs) include a large group of viruses of at least 12 genera, nine families, and two orders (Shi et al., 2018 ). In China, tick-borne encephalitis virus (TBEV) in the family *Flaviviridae* and Crimean-Congo hemorrhagic fever virus (CCHFV) in the family *Nairoviridae* are responsible for encephalitis and hemorrhagic fever in northeastern and northwestern China, respectively. Emerging TBVs, such as severe fever with thrombocytopenia virus (Yu et al., 2011), Jingmen tick virus (Jia et al., 2019), Alongshan virus (Wang et al., 2019), Songling virus (Ma et al., 2021), Beiji nairovirus (Wang et al., 2021), and Tacheng tick virus 1 and 2 (Liu et al., 2020; Dong et al., 2021), have been reported to be associated with human diseases. In addition, the Nairobi sheep disease virus, another member in the family *Nairoviridae* that can cause acute hemorrhagic gastroenteritis in sheep and goats, has also been found in ticks in China (Gong et al., 2015).

High-throughput sequencing technology has been widely used to investigate viromes in ticks and diagnose unknown infectious diseases. To date, tick viromes have been reported in Heilongjiang, Liaoning, Hebei, Henan, Hubei, and Yunnan provinces of China (Meng et al., 2019; Zhao et al., 2020; Shi et al., 2021; Xu et al., 2021; Yang et al., 2021), revealing different tick viromes, due to tick species and geographical differences. Guangdong province of China is considered as one of the global hotspots of emerging zoonotic diseases and vector-borne diseases resulting from the specific climate, environment, and biodiversity (Wang et al., 2008; Allen et al., 2017), but the viromes in ticks in Guangdong, southern China remain poorly understood. In this study, we identified RNA viruses in *Rhipicephalus sanguineus* and *Rhipicephalus microplus* ticks in Guangdong, southern China using metagenomics and detected 10 viruses, including 3 novel viruses, suggesting a high viral diversity in *Rhipicephalus* ticks.

## Materials and methods

### Sample collection, processing, and RNA extraction

From May–June 2020, ticks were collected from cattle and dogs in Zhanjiang and Jieyang, Guangdong, southern China. Tick species were morphologically identified by an experienced technician and confirmed by sequencing the mitochondrial 16S ribosomal RNA (16S rRNA) gene (Liu et al., 2016). The sampled ticks were divided into 6 groups according to the species and collection location. After washing with phosphate buffered saline (PBS), ticks were homogenized in PBS solution, followed by centrifugation at 2,500 × *g* for 5 min at 4°C. Supernatants were collected for RNA extraction with TRIzol LS reagent (Invitrogen, Carlsbad, CA, United States).

### Meta-transcriptomics and bioinformatics analyses

Meta-transcriptome sequencing was conducted as previously described (Shi et al., 2016). In brief, after removing ribosomal RNA (rRNA), RNA was fragmented, reverse-transcribed, and adapted, followed by paired-end (150-bp) sequencing on the Illumina Hiseq 2,500 platform. All library preparation and sequencing were performed by Tianjin Novogene Bioinformatics Technology Co., Ltd., Tianjin, China.

Sequencing reads were adaptor- and quality-trimmed using the FASTP program, followed by *de novo* assemble by the Megahit program, and the resulting contigs were compared against the nr database using the diamond BLASTX program. The confirmed viral contigs with assembly overlaps or from the same scaffold were merged using the SeqMan program (version 7.1, DNAstar, Madison, WI, United States). Reads were mapped to the target contigs using Bowtie 2, and the integrated genomics viewer (IGV) was used to check for assembly faults in order to validate the assembly results. Gaps between the contigs were filled by RT-PCR and Sanger sequencing. The genomic terminus of targeting viruses was determined using the 5′/3′ RACE kits (TaKaRa, Dalian, China). The complete viral genome was confirmed by Sanger sequencing with overlapping primers that covered the entire viral genome.

### Virus classification

The discovered viruses were classified based on the nucleotide (nt) and amino acids (aa) identities. If the species demarcation criteria remain unclear within a genus, a novel viral species is defined if it holds less than 80% nt identity across the complete genome or less than 90% aa identity of the RNA-dependent RNA polymerase (RdRp) domain with known viruses. All the novel viruses were named “Guangdong tick,” followed by common viral names according to their taxonomy. Viruses classified into established taxonomies were marked with “Guangdong (GD)” to distinguish them from other viral strains.

### Phylogenetic analysis

Reference virus sequences were acquired from the GenBank database and examined using Mafft v7.450 to determine the phylogenetic relationships of the identified viruses (Katoh et al., 2019). Phylogenetic trees were constructed using the maximum-likelihood method in PhyML v3.0 with Le and Gascuel substitution model for aa sequence analysis and General Time Reversible substitution model for nt sequence analysis, based on a bootstrap value of 1,000 replicates (Guindon et al., 2005).

## Results

### Viral diversity in ticks in Guangdong

A total of 339 ticks were collected from cattle (*n* = 306) and dogs (*n* = 33) in Jieyang (*n* = 242) and Zhanjiang (*n* = 97) of Guangdong Province, China, including 243 *R. sanguineus* and 96 *R. microplus* (Supplementary Table S1; Supplementary Figure S1). Ticks were pooled into 6 groups for RNA library construction and sequencing. After quality control and adapter trimming, a total of 85,275,714 paired-end clean reads were generated in these libraries, resulting in 259,311 viral reads, which accounted for 0.30% of the total RNA reads, and assembled to 190 viral contigs (Supplementary Tables S2, S3). After being aligned by Blast, viral contigs were finally annotated to 10 viruses of six known viral families of *Phenuiviridae*, *Chuviridae*, *Flaviviridae*, *Reoviridae*, *Orthomyxoviridae*, *Rhabdoviridae*, and an unclassified family (Supplementary Table S4).

### Viral genome organization and phylogeny characterization

The complete genomes of representative 23 viral strains were obtained by contig-based PCR and RACE (Supplementary Table S5), including 2 novel viruses, such as Guangdong tick Manly virus (GTMV) and Guangdong tick quaranjavirus (GTQV), 7 known viruses, including Brown dog tick phlebovirus 1 (BDTPV1), Lihan tick virus (LTV), Mivirus sp. (MIV), Wuhan tick virus 1 (WTV1), Hepacivirus N (HNV), Wuhan tick virus 2 (WTV2) and Brown dog tick phlebovirus 2 (BDTPV2) and a virus with partial sequence named Guangdong tick orbivirus (GTOV; Supplementary Table S6). The mean depth of viral sequencing in each pool is shown in Supplementary Table S7, and these viruses were named based on the viral species, the pool name they discovered, and the closest relationships with previously described tick-associated viruses.

### *Phenuiviridae*: LTV GD, BDTPV1 GD, and BDTPV2 GD

Metagenomic analysis demonstrated that bunyavirus reads accounted for 51.4% (133,381/259311) of total viral reads, including 1.04% (2,721/259311), 22.89% (59,367/259311), and 27.49% reads (71,293/259311) mapped to the genomes of LTV, BDTPV1, and BDTPV2, respectively (Supplementary Tables S3, S8). We obtained 10 complete viral genomes and designated LTV GD (JY02-3 and ZJ02), BDTPV1 GD (JY01, JY02-1, JY02-2, JY02-3, and ZJ-0103) and BDTPV2 GD (JY01, JY02-1, and JY02-3; Supplementary Table S4).

**Figure 1:**
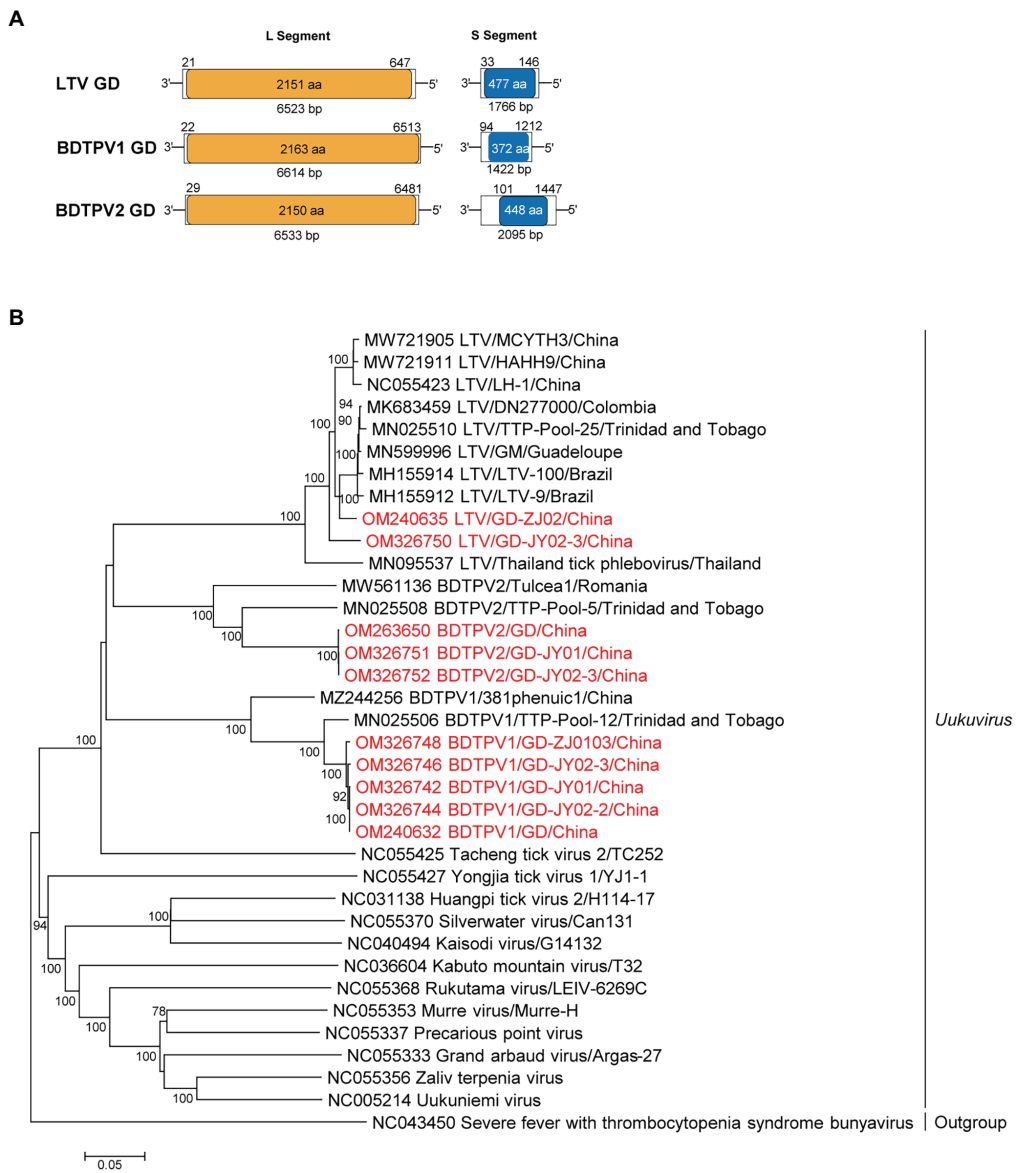
The genome characterization and phylogenetic analysis of uukuviruses. **(A)** Genome organization and putative coding regions of Lihan tick virus (LTV), Brown dog tick phlebovirus 1 (BDTPV1) and 2 (BDTPV2). The viral genome includes L and S segments that contained the predicted RNA depended RNA polymerase (RdRp) and nucleoprotein (NP) respectively. **(B)** Phylogenetic analyses of uukuviruses. Phylogenetic trees were constructed based on the L segment nucleotide sequences of representative viruses in the genus *Uukuvirus*. Viruses obtained in ticks here are highlighted in red. Scale bar indicates nucleotide substitutions per site.

The genomes of these viruses contained bi-segments, large (L) and small (S) segments, encoding the putative RdRp and nucleoprotein, respectively (Figure 1). As shown in Supplementary Figure S1; Supplementary Table S8, the S segment of LTV GD contains a 1766 bp putative nucleocapsid ORF, whereas the L segment encodes a 6,614-bp RdRp. The amino acid sequences of LTV GD L proteins were approximately 97.5–99.3%, similar to the L proteins of other LTVs (Figure 1; Supplementary Table S8). The length of L and S segments for BDTPV1 were 6,614 bp and 1,422 bp, sharing 98.9–99.4% aa identity in the RdRp with BDTPV1 strain TTP-Pool-12, which has been reported in Trinidad and Tobago (Sameroff et al., 2019). The length of L and S segments for BDTPV2 was 6,533 bp and 2095 bp. BDTPV2 GD shared 96.7% aa identity with BDTPV2 strain TTP-Pool-5 in the RdRp (Sameroff et al., 2019). Interestingly, both BDTPV1 and BDTPV2 were found to co-exist in five *R. sanguineus* tick pools.

Phylogenetic analyses revealed that all three identified viruses clustered with the other bunyaviruses lacking M segment and shared the common ancestor with the *Uukuvirus* group. They were genetically related to those identified in Brazil, Thailand, Colombia, or Trinidad and Tobago (Figure 1).

### *Chuviridae*: MIV GD and WTV2 GD

In total, 39,371 reads (15.18%; 39,371/259311) from 3 *R. sanguineus* tick pools were mapped to the proposed *Mivirus*, while 20,296 reads (7.83%; 20,296/259311) in 3 pools were matched with WTV2 (Supplementary Table S3).

**Figure 2:**
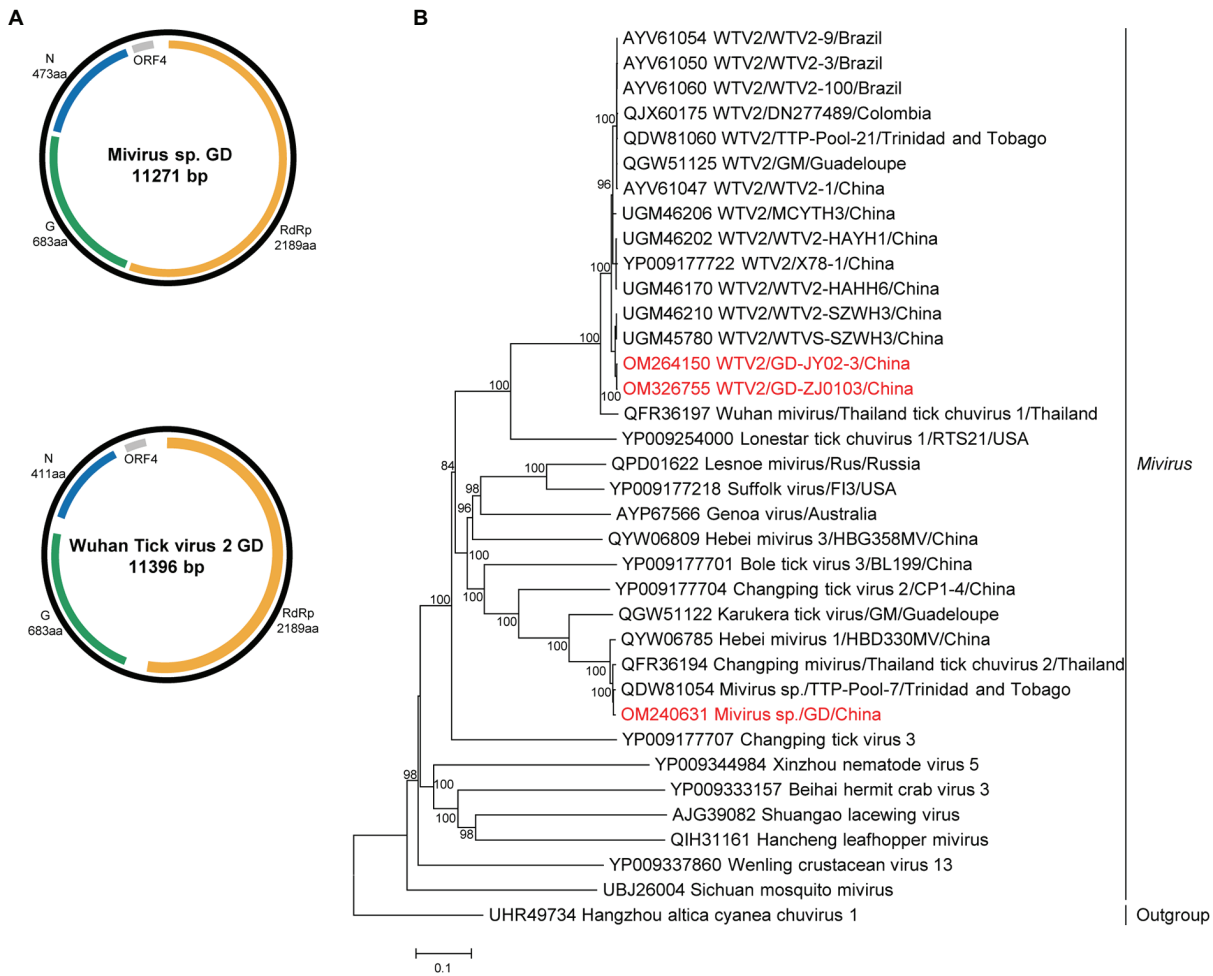
The genome structure and phylogenetic analysis of miviruses. **(A)** Genome organization and putative coding regions of Mivirus sp. (MIV) and Wuhan tick virus 2 (WTV2). The viral genome contains one segments that encoding the RdRp, glycoprotein (G), nucleoprotein (N) and ORF4. **(B)** Phylogenetic analyses of miviruses. Phylogenetic trees were constructed based on the RdRp protein sequences of representative viruses in the genus *Mivirus*. Viruses obtained in ticks here are highlighted in red. Scale bar indicates nucleotide substitutions per site.

The complete genomes of miviruses were obtained and named MIV GD and WTV2 GD JY02-3 and ZJ2103. Both the WTV2 and MIV genome of the study were confirmed to belong to the circular structure by performing PCR with the forward primer located in 3′ of the genome and the reverse primer in the 5′ part (Supplementary Table S5). The genome included three ORFs (Figure 2), encoding the putative RdRp, glycoprotein, and nucleoprotein, respectively. The length of MIV GD was 11,271 bp, with the high identity (99.5% of RdRp aa sequence, 97.8% of complete nt sequence) to MIV strain TTP-Pool-7 discovered in Trinidad and Tobago (Sameroff et al., 2019). The length of WTV2 GD (JY02-3 and ZJ2103 strain) were 11,396 bp, sharing 99.5–99.6% aa identity in the RdRp with the strain WTVS-SZWH3 identified in China (Xu et al., 2021; Supplementary Table S9).

Phylogenetically, both chuviruses identified in this study were grouped into the Mivirus clade and closely related to the viral strains discovered in other countries, such as Brazil, Thailand, Colombia, or Trinidad and Tobago (Figure 2).

### *Rhabdoviridae*: WTV1 GD

**Figure 3:**
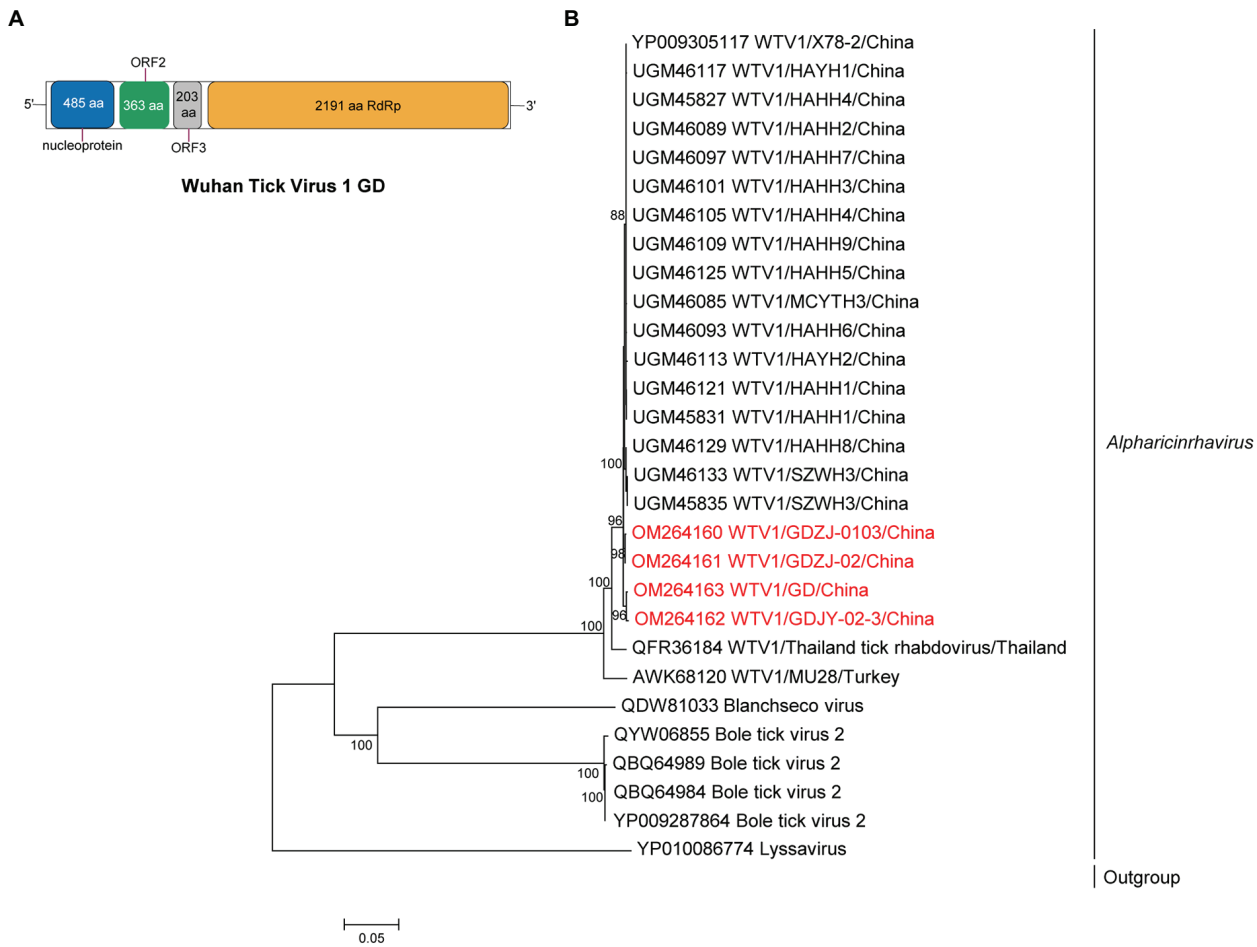
The genome structure and phylogenetic analysis of alpharicinrhaviruses. **(A)** Genome organization and putative coding regions of Wuhan tick virus 1 (WTV1). The viral genome contains one segments that encoding the nucleoprotein (N), ORF2, ORF3, and RdRp. **(B)** Phylogenetic analyses of alpharicinrhaviruses. Phylogenetic trees were constructed based on the RdRp protein sequences of representative viruses in the genus *Alpharicinrhavirus*. Viruses obtained in ticks here are highlighted in red. Scale bar indicates nucleotide substitutions per site.

Metagenomics revealed 3.42% of viral reads (8,861/259311) mapped to WTV1 (Supplementary Table S3), and four complete genomes of WTV1 were obtained and designated WTV1 GD strains ZJ0103, ZJ02, JY02-1, and JY02-3. The full length of WTV1 GD was 10,174 bp, which shared 95.7–99.7% aa identity of RdRp with other strains in China (Supplementary Table S10). WTV1 GD exhibited a non-classic rhabdo-like genome organization, with four open reading frames (ORFs), encoding RdRp, nucleoprotein (N), hypothetical protein 2 (ORF2) and 3 (ORF3). However, the G gene was not found in WTV1 GD (Figure 3). Phylogenetic analysis showed WTV1 had a certain regional difference, though Chinese WTV1 GD strains were clustered with the viral strains identified in Thailand and Turkey (Figure 3).

### *Flaviviridae*: BovHepV

Approximately 6.1% of reads (233/3642) in GDZJ/02 collected from cattle in Zhanjiang were assigned to BovhepV in the genus *Hepacivirus* in the family *Flaviviridae* (Supplementary Table S3). Three strains of BovhepV shared 84.4–84.8% nt identity and 94.8–95.6% aa identity with Brazil strains and formed a novel subtype in genotype 1 (Shao et al., 2021).

### *Orthomyxoviridae*: GTQV

**Figure 4:**
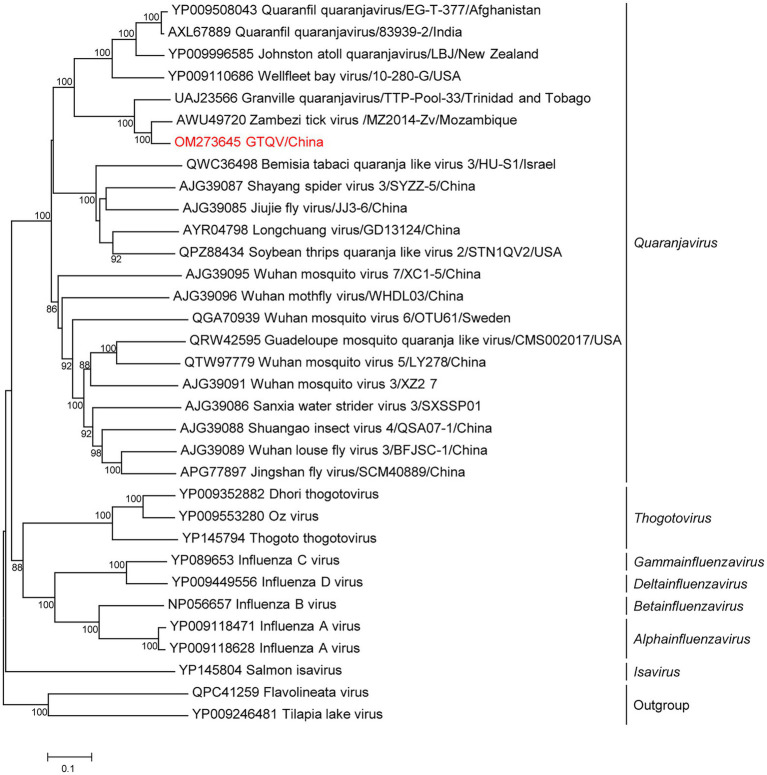
Phylogenetic analysis of orthomyxoviruses. Phylogenetic trees of Guangdong tick quaranjavirus (GTQV) were constructed based on the polymerase basic protein 1 (PB1) protein sequences of representative viruses in the family *Orthomyxovirus*. Viruses obtained in ticks here are highlighted in red. Scale bar indicates nucleotide substitutions per site.

Metagenomics showed that 56,128 reads (21.65%; 56,128/2,59,311) in five pools were annotated to GTQV (Supplementary Table S3). We identified a quaranja-like virus in five pools, tentatively named Guangdong tick quaranjavirus (GTQV). The complete genome of GTQV included six segments, PB2, PB1, PA, NP, HA and M, ranging from 930 to 2,422 bp. The VP7 segment of quaranjavirus was likely to be absent in GTQV (Figure 4).

GTQV shared 57.4–86.2% aa identity to the quaranjaviruses (Supplementary Table S11), whose PB1 had 86.2 and 72.1% aa identity to Zambezi tick virus 1 (Cholleti et al., 2018) and Granville quaranjavirus, respectively (Sameroff et al., 2021). The putative M segment had a similar length to other quaranjaviruses, including two open reading frames (ORF; Supplementary Figure S2). ORF1 had the start codon ATG and encoded a 154-aa protein (Supplementary Figure S3); ORF2 had the start codon GTG and encoded a 79-aa protein. The GTQV genomic segments had a conserve 5′-terminal sequence UGCUGUGGCUGC and 3′-terminal sequence GCTTGTCTCTACT.

Phylogenetic analysis of the complete PB1 protein showed that GTQV was clustered with quaranjaviruses in the family *Orthomyxoviridae*. Compared with other quaranjaviruses, the GTQV genome had a specific terminal sequence (Supplementary Figure S3).

### *Reoviridae*: GTOV

Metagenomic analysis showed that 391 reads (0.15%, 391/259311) were mapped with the reovirus-like virus in the *R. microplus* pool of Zhanjiang (Supplementary Table S3). The complete ORF of six segments (VP4, VP5, VP6, NS1, NS2, and NS3) and partial ORF of three segments (VP1, VP2, and VP3) were obtained. Unfortunately, the VP7 gene could not be found in our study. We designated this virus as Guangdong tick orbivirus (GTOV), whose genome shared 73.1–80.5% nt and 70–88.1% aa identity with the known reoviruses (Supplementary Table S4).

Phylogenetic analysis showed that GTOV was genetically related to the known reoviruses (Supplementary Figure S4), in which VP1, VP4 and NS3 were closely related to the Wad Medani virus SUD1952/01 identified in *Rhipicephalus* in Sudan (Belaganahalli et al., 2015). VP3, VP5, VP6 and NS1 were closely related to the strain Tuva 2012 in Russia (Dedkov et al., 2021), and VP2 was closely related to the strain LEIV-8066Tur in Tajikistan, while NS2 was closely related to the isolate G-673 in India (Yadav et al., 2018).

### Unclassified mononegavirus: GTMV

Mainly virus, first discovered in ticks fed on reptiles in Australia, is an unclassified mononegavirus (Harvey et al., 2019). In this study, a total of 860 (0.33%, 391/259,311) reads were mapped with the mainly virus in pools of Jieyang. We named this virus as Guangdong tick mainly virus (GTMV), which genome had 10,177 nt and shared 44.9% nt and 36.5–65.5% aa similarity with mainly virus.

## Discussion

This study reported the metagenomic analysis of RNA viruses in ticks in Guangdong, southern China, revealing a high viral diversity in these collected ticks and 10 different viruses belonging to six viral families of *Phenuiviridae*, *Chuviridae*, *Flaviviridae*, *Reoviridae*, *Orthomyxoviridae*, and *Rhabdoviridae* and unclassified family, were identified. These viral families were similar to those described in Turkey (Brinkmann et al., 2018), Trinidad and Tobago (Sameroff et al., 2019), Australia (Harvey et al., 2019), Thailand (Temmam et al., 2019), northern Europe (Pettersson et al., 2017), southern Brazil (Souza et al., 2018), and Heilongjiang, Yunnan, and Zhejiang provinces of China (Meng et al., 2019; Shi et al., 2021; He et al., 2022), which may provide the evidence of long-distance spread of tick-borne viruses in Guangdong, southern China through migratory birds or animal movement.

The family *Chuviridae* includes at least 14 viral genera, genetically located between the segmented and the unsegmented negative-sense RNA viruses. *Mivirus*, one genus in the family *Chuviridae*, contained at least 9 viral species discovered in ticks. MIV GD was genetically grouped into the miviruses, closely related to the MIV strain TTP-Pool-7 (Sameroff et al., 2019). Mivirus discovered in this study had a closer relationship with the strains discovered in south America than those identified in China. Wuhan tick virus 2 (WTV2) was firstly discovered in *R. microplus* ticks from Wuhan, China, and then discovered in South America (Li et al., 2015; Souza et al., 2018; Sameroff et al., 2019; Gomez et al., 2020). WTV2 GD and WTV2-X78-1 were clustered with the strains discovered in South America (Li et al., 2015), but had a higher identity than the strains identified in China. These results confirmed the long-distance viral transmission, but the pathogenicity of MIV GD and WTV2 GD needs further investigation.

The family *Orthomyxoviridae* is composed of seven genera: types A/B/C and D *Influenzavirus*, *Isavirus*, *Quaranjavirus*, and *Thogotovirus*. Tick-borne orthomyxoviruses are mainly located in the genus *Quaranjavirus* and *Thogotovirus*. The first quaranjavirus was isolated from soft ticks and the blood of children suffering from an unexplained febrile illness (Taylor et al., 1966). Quaranjaviruses are also associated with mass avian die-offs (Allison et al., 2015). GTQV identified here was clustered with other recently identified hard-tick-associated quaranjaviruses, with wide geographic ranges, suggesting that many unidentified novel tick-borne orthomyxoviruses were classified based on the phylogenetic analysis.

LTV was first described in Chinese *R. microplus* (Li et al., 2015) and reported in Turkish *H. marginatum* and *R. sanguineus* ticks (Dincer et al., 2017; Brinkmann et al., 2018), and Colombian *Dermacentor nitens* ticks (Lopez et al., 2020). LTV GD was closely related to the variants found in China (Xu et al., 2021), France (Gondard et al., 2020), and Brazil (Souza et al., 2018). The discovery of LTVs in various tick species worldwide suggests that it might present a low degree of host restriction. In addition, based on the co-presence of BDTPV1 and BDTPV2 in all five *R. Sanguineus* tick pools, consistent with a study in Latin America (Sameroff et al., 2019), we hypothesized that BDTPV1 and BDTPV2 may extensively co-infect *R. Sanguineus* or had a symbiotic relationship with *R. Sanguineus*. Analysis of additional tick species from different geographic regions will help confirm the evolutionary association of these viruses with their tick hosts.

Rhabdoviruses can infect a wide range of hosts, including vertebrates, arthropods and plants, and the family has 3 subfamilies (*Alpharhabdovirinea*, *Betarhabdovirinae*, and *Gammarhabdovirinae*). *Alpharicinrhavirus* is a genus of *Alpharhabdovirinea,* containing three species of viruses discovered in ticks. Wuhan tick virus 1 (WTV1), belonging to the *Alpharhabdovirinea* subfamily, was first detected in *R. microplus* from Wuhan, China (Li et al., 2015). WTV1 GD and other reported strains clustered together, showing the conservation of genetic evolution in geographical locations.

Reoviruses have been identified in ticks for decades, in which the Colorado tick fever virus is responsible for an acute systemic febrile illness in humans in the United States and Canada (Klasco, 2002). However, tick-borne reovirus remains to be determined in China. GTOV identified in *R. microplus* shared 84.6% polymerase and 78.4% inner core protein identity to the Wad Medani virus, suggesting GTOV may represent a novel reovirus species. However, the pathogenicity of GTOV needs to be further investigated. GTOV, first reported in China, showed a close relationship with available WMV strains isolated from *Rhipicephalus* in Sudan in 1952 (Liu et al., 2016). Previous high-throughput sequencing studies on ticks in China have failed to detect the virus. It is possible that GTOV is not a native virus but instead transmitted to China through livestock trade or migratory birds. Moreover, intersegment reassortment is likely to be as a source of genetic diversity in tick-borne orbiviruses, and multiple reassortment events have also been found within the WMV group.

We also detected bovine hepacivirus in the family *Flaviviridae* in ticks collected from cattle, where ticks may be the mechanical carrier (Shao et al., 2021). Further investigation is needed to confirm the pathogenicity of bovine hepacivirus and possible transmission routes of bovine hepacivirus.

## Conclusion

This study investigated the virome of *Rhipicephalus* ticks in Guangdong, southern China, and detected 10 viruses, including Lihan tick virus, Brown dog tick phlebovirus 1 and 2 in the family *Phenuiviridae*, Mivirus and Wuhan tick virus 2 in the family *Chuviridae*, Wuhan tick virus 1 in the family *Rhabdoviridae*, Hepacivirus N in the family *Flaviviridae*, Guangdong tick quaranjavirus (GTQV) in the family *Orthomyxoviridae*, Guangdong tick orbivirus (GTOV) in the family *Reoviridae*, and Guangdong tick Manly virus (GTMV) of an unclassified family, in which most of them were genetically related to the virus strains in countries outside China, and GTQV, GTOV, and GTMV may represent novel viral species. These findings provided the evidences of the long-distance spread of these TBVs in Guangdong, southern China, suggesting the necessity and importance of TBV surveillance.

## Data availability statement

The datasets presented in this study can be found in online repositories. The names of the repository/repositories and accession number(s) can be found in the article/Supplementary material.

## Author contributions

ZW and QL conceived the project. JM, JL, WH, LZ, and KZ collected the samples. LG, MC, WL, JZ, QJ, HH, and LZ conducted experiment. LG and QL analyzed the data and drafted the manuscript. All authors were involved in critically revised the manuscript and approved the final version.

## Funding

The study was funded by the Science and Technology Innovation Project in Foshan, Guangdong Province, China (2020001000151), the Pearl River Talent Plan in Guangdong Province of China (2019CX01N111), and Guangzhou Science and Technology Project of China (202103000008).

## Conflict of interest

The authors declare that the research was conducted in the absence of any commercial or financial relationships that could be construed as a potential conflict of interest.

## Publisher’s note

All claims expressed in this article are solely those of the authors and do not necessarily represent those of their affiliated organizations, or those of the publisher, the editors and the reviewers. Any product that may be evaluated in this article, or claim that may be made by its manufacturer, is not guaranteed or endorsed by the publisher.
