# Supplementary material for: Virome of Rhipicephalus ticks by metagenomic analysis in Guangdong, southern China
Source: Front Microbiol. 2022 Aug 11;13:966735. doi: 10.3389/fmicb.2022.966735 (PMC9403862; doi:10.3389/fmicb.2022.966735)
Supplement: Supplementary file 1 [file Data_Sheet_1.doc]

# Virus diversity in *Rhipicephalus* ticks revealed by metagenomic analysis in Guangdong, Southern China

Supplementary materials


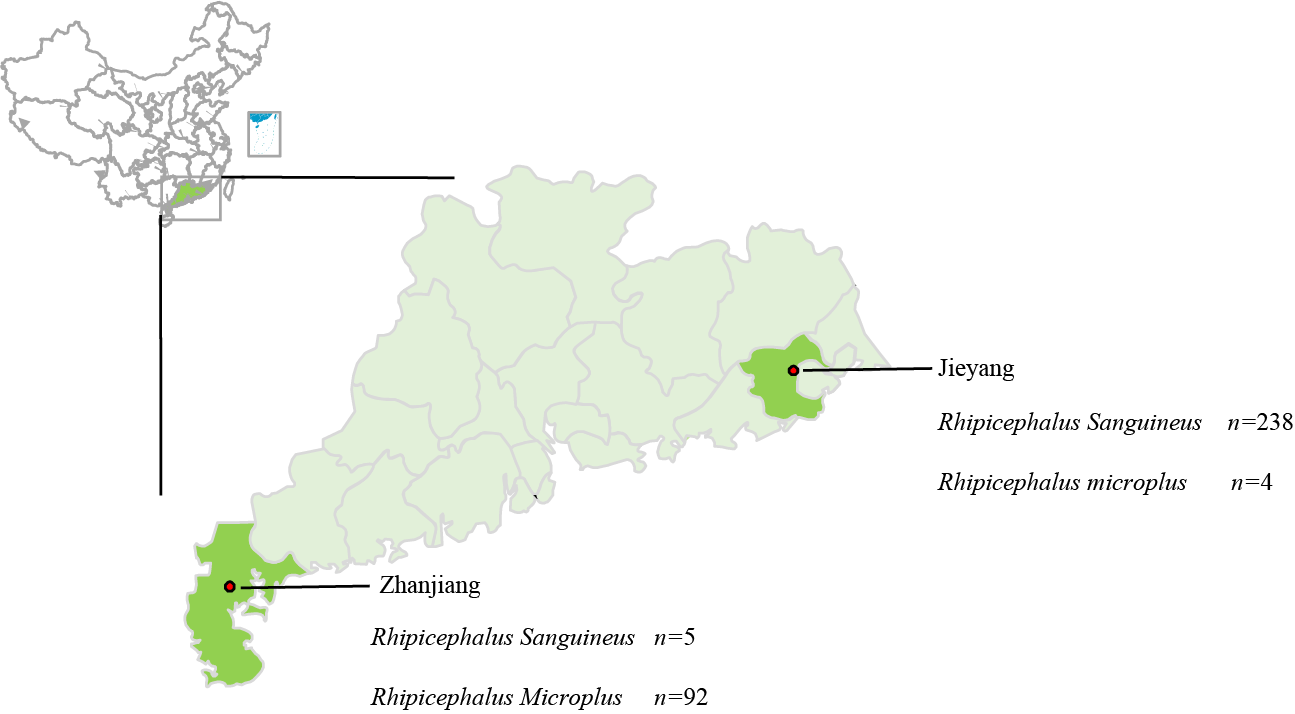


**Supplementary Fig. S1.** Tick samples were collected from Zhanjiang and Jieyang in Guangdong, southern China. Sampling sites are marked by red dot and the tick species and number are shown.

**

**

**Supplementary Fig. S2.** Schematic representation, coverage, and annotation of genomes of RNA viruses found in ticks in Guangdong. Y axis represents the sequencing depth.

**
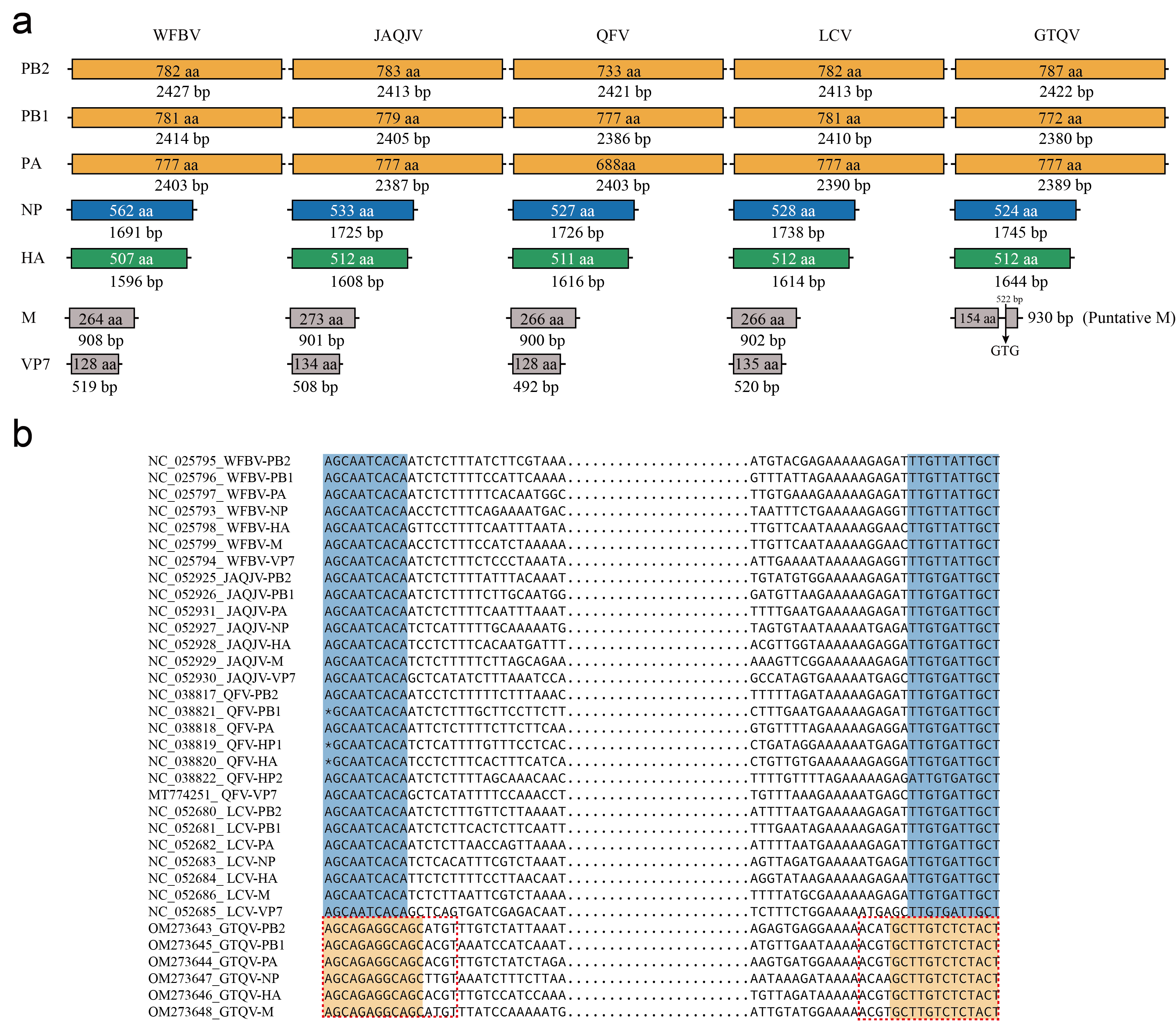
**

**Supplementary Fig. S3.** Genomic comparison of Guangdong tick quaranjavirus (GTQV) and other identified Quaranjaviruses. (**a)** Genomic illustration of GTQV and Quaranjaviruses. The predicted ORFs are annotated with different colors: RdRp subunit protein (PB2, PB1, PA) shaded with yellow, nucleoprotein (NP) is indicated with blue, hemagglutinin protein (HA) is presented with green, and the matrix protein (M) and other hypothetical viral protein (VP7) is showed with gray. (**b)** The terminal sequences of GTQV and other Quaranjaviruses. The typical terminal sequences of Quaranjaviruses are marked by blue boxes and the conserved GTQV terminal sequences are marked with yellow boxes. The sequences that have a reverse-complementary sequence at the other terminal in the same segment are marked with a frame of red dot line. Abbreviations: WFBV, Wellfleet Bay virus strain 10-280-G strain; JAQJV, Johnston Atoll quaranjavirus strain LBJ; QFV, Quaranfil virus strain EG T-377; LCV, Lake Chad virus isolate Ib An 38918; GTQV, Guangdong tick quaranjavirus; HP, hypothetical protein; *, the terminal position which may lack a A nucleotide.


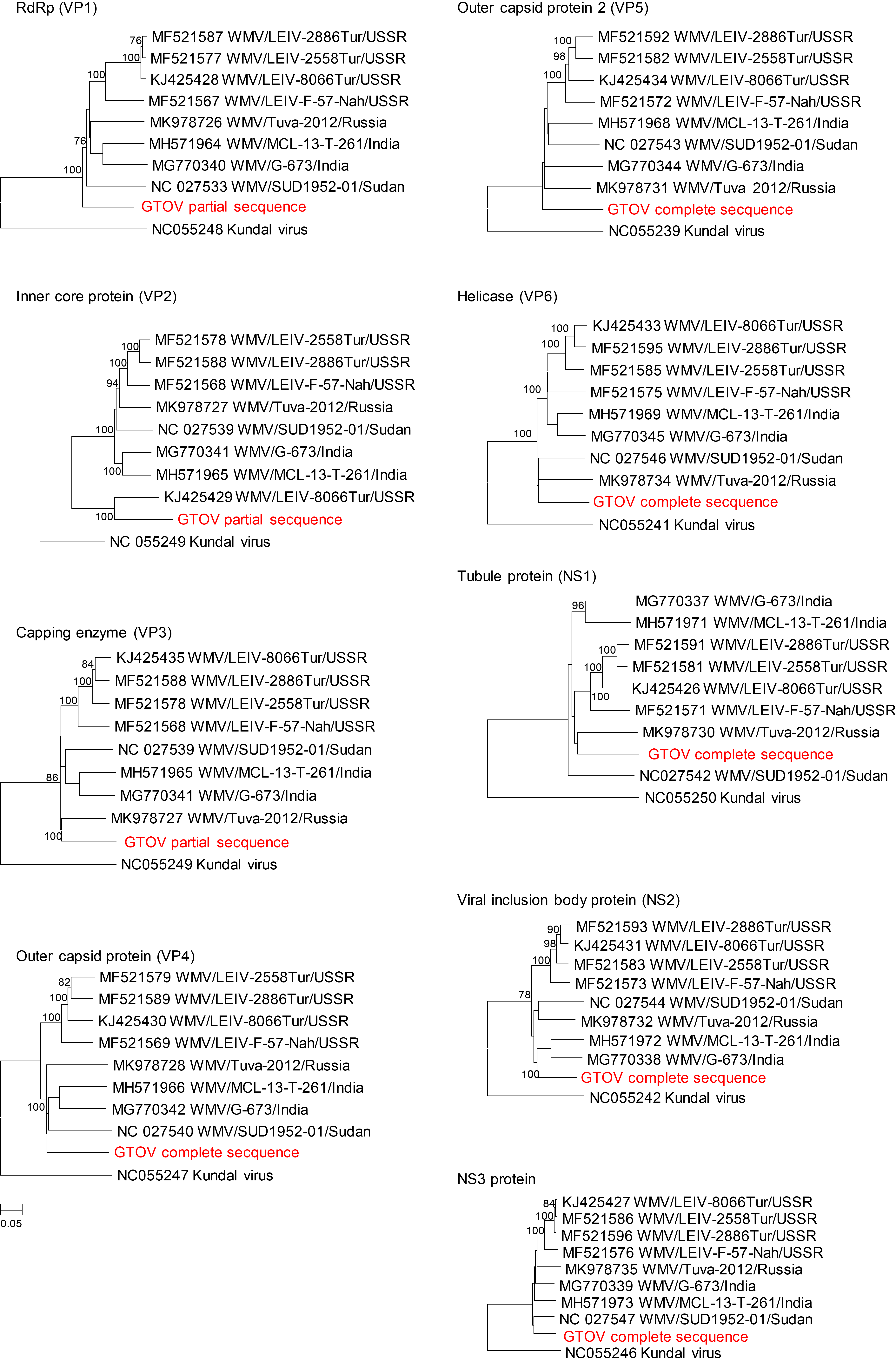


**Supplementary Fig. S4.** Phylogenetic analyses of Wad Medani virus (WMV) and Guangdong tick orbivirus (GTOV). Phylogenetic trees were constructed based on the nucleotide sequence of viruses. Viruses obtained in ticks here are highlighted in red.

**Supplementary Table S1.** Ticks collected in Guangdong province, Southern China

| **Regions** | **Tick species** | **Tick host** | **No. of collected ticks** |
| --- | --- | --- | --- |
| Jieyang | *Rhipicephalus sanguineus* | Cattle | 238 |
|  | *Rhipicephalus microplus* | Cattle | 4 |
| Zhanjiang | *Rhipicephalus sanguineus* | Dogs | 5 |
|  | *Rhipicephalus microplus* | Cattle | 92 |
| Total | | | 339 |

**Supplementary Table S2.** Summary of sampling, pooling and RNA sequencing

| **Pooling** |  |  |  |  |  | **No. of reads by RNA sequencing** |  |  |
| --- | --- | --- | --- | --- | --- | --- | --- | --- |
| **Sampling location** | **Name of pooled ticks** | **Tick Species*** | **No. of ticks** | **Tick host** | **Total reads of RNA sequencing** | **Total viral reads** | **Percentage of viral reads** | **Viral contigs** |
| GDJY/01 | Jieyang | *R. sanguineus* | 88 | Cattle | 8874174 | 50640 | 0.57% | 13 |
| GDJY/02/1 |  | *R. sanguineus* | 76 | Cattle | 20064646 | 140181 | 0.70% | 34 |
| GDJY/02/2 |  | *R. sanguineus* | 53 | Cattle | 13743304 | 32929 | 0.24% | 31 |
| GDJY/02/3 |  | *R.microplus R. sanguineus* | 4  21 | Cattle Cattle | 24040954 | 20691 | 0.09% | 24 |
| GDZJ/02 | Zhanjiang | *R.microplus* | 63 | Cattle | 10627704 | 3642 | 0.03% | 50 |
| GDZJ/0103 |  | *R.microplus R. sanguineus* | 5  29 | Cattle Dogs | 7924932 | 11228 | 0.14% | 38 |
|  | Total |  | 339 |  | 85275714 | 259311 | 0.30% | 190 |

**Supplementary Table S3.** Number of viral reads in each pool

Abbreviations: BDTPV1 GD, Brown dog tick phlebovirus 1 Guangdong; LTV GD, Lihan tick virus GD; MIV GD, Mivirus sp. GD; WTV1 GD, Wuhan tick virus 1 GD; HNV GD, Hepacivirus N GD; HTV2 GD, Wuhan tick virus 2 GD; BDTPV2 GD, Brown dog tick phlebovirus 2 GD; GTOV, Guangdong tick orbivirus; GTQV, Guangdong tick quaranjavirus; GTMV, Guangdong tick Manly virus.

|  | **Name of pooled ticks** | | | | | |
| --- | --- | --- | --- | --- | --- | --- |
| **Virus name** | **GDJY/01** | **GDJY/02/1** | **GDJY/02/2** | **GDJY/02/3** | **GDZJ/02** | **GDZJ/0103** |
| BDTPV1 GD | 7644 | 39298 | 5733 | 712 | 0 | 5980 |
| BDTPV2 GD | 16393 | 34989 | 14299 | 3966 | 0 | 1646 |
| LTV GD | 0 | 0 | 0 | 1593 | 868 | 260 |
| MIV GD | 19938 | 18611 | 822 | 0 | 0 | 0 |
| WTV2 GD | 0 | 9011 | 0 | 10616 | 0 | 669 |
| WTV1 GD | 0 | 921 | 0 | 3297 | 2160 | 2283 |
| HNV GD | 0 | 0 | 0 | 0 | 223 | 0 |
| GTQV | 6665 | 36745 | 12075 | 253 | 0 | 390 |
| GTOV | 0 | 0 | 0 | 0 | 391 | 0 |
| GTMV | 0 | 606 | 0 | 254 | 0 | 0 |
| Total identified virus reads | 50640 | 140181 | 32929 | 20691 | 3642 | 11228 |

**Supplementary Table S4.** Information of viral genome obtained in this study

| **Virus name** | **Viral family** | **Genetically closest virus** | **GenBank accession number of genetically closest virus** | **Genome segment** | **Length of the gene (bp)** | **Identity with relative virus (nt, %)** | **Protein*** | **Identity with relative virus (aa, %)** |
| --- | --- | --- | --- | --- | --- | --- | --- | --- |
| BDTPV 1 GD | *Phenuiviridae;Uukuvirus* | Brown dog tick phlebovirus 1 | MN025506 | L | 6614 | 95.39 | RdRp | 99.3 |
|  |  |  | MN025507 | S | 1422 | 98.52 | nucleoprotein | 98.6 |
| BDTPV 2 GD | *Phenuiviridae;Uukuvirus* | Brown dog tick phlebovirus 2 | MN025508 | L | 6533 | 84.8 | RdRp | 96.0 |
|  |  |  | MN025509 | S | 2095 | 90.43 | nucleoprotein | 91.5 |
| LTV GD | *Phenuiviridae;Uukuvirus;* | Lihan tick virus | MN599996 | L | 6523 | 96.03 | RdRp | 99.2 |
|  |  |  | MN599997 | S | 1766 | 95.73 | nucleoprotein | 97.7 |
| MIV GD | *Chuviridae;Mivirus* | Mivirus sp. | MN025520 | ssRNA(-) | 11271 | 97.7 | RdRp | 99.4 |
|  |  |  |  |  |  |  | glycoprotein | 99.0 |
|  |  |  |  |  |  |  | nucleoprotein | 98.9 |
|  |  |  |  |  |  |  | hypothetical protein | 100 |
| WTV2 GD | *Chuviridae;Mivirus* | Wuhan tick virus 2 | MW721953 | ssRNA(-) | 11396 | 97.7 | RdRp | 97.4 |
|  |  |  |  |  |  |  | glycoprotein | 96 |
|  |  |  |  |  |  |  | nucleoprotein | 97.1 |
|  |  |  |  |  |  |  | hypothetical protein | 93.5 |
| WTV1 GD | *Rhabdoviridae;* | Wuhan Tick Virus 1 | MW721927 | ssRNA(-) | 10174 | 96.6 | nucleocapsid | 99.0 |
|  | *Alpharicinrhaviru*s |  |  |  |  |  | ORF2/hypothetical protein | 97.2 |
|  |  |  |  |  |  |  | ORF3/hypothetical protein | 97.5 |
|  |  |  |  |  |  |  | RdRp | 99.4 |
| HNV GD | Flaviviridae;Hepacivirus | Hepacivirus N | MG781019 | ssRNA(+) | 8808 | 85.1 | polyprotein | 95.6 |
| GTQV | *Orthomyxoviridae;* | Zambezi tick virus 1 | MH267793 | PB1 | 2380 | 76.7 | polymerase basic 1 protein | 86.2 |
|  | *Quaranjavirus* | Granville quaranjavirus | MZ502308 | PB2 | 2422 | 65.9 | polymerase basic 2 protein | 57.4 |
|  |  | Granville quaranjavirus | MZ502307 | PA | 2389 | 64.4 | polymerase acidic protein | 66.0 |
|  |  | Granville quaranjavirus | MZ502306 | HA | 1644 | 64.4 | hemagglutinin protein | 65.1 |
|  |  | Granville quaranjavirus | MZ502304 | NP | 1745 | 60.9 | nucleoprotein | 60.8 |
|  |  | NS | NS | M | 930 | NS | matrix protein | NS |
| GTOV | *Reoviridae;Orbivirus* | Wad Medani virus | MF521567 | VP1 | 1693^p^ | 74.50^p^ | RdRp | 84.6^p^ |
|  |  |  | MF521590 | VP2 | 1714^p^ | NS | outer capsid protein | 84.6^p^ |
|  |  |  | MF521588 | VP3 | 1493^p^ | 78.35^p^ | inner core protein | 78.4^p^ |
|  |  |  | KJ425430 | VP4 | 1941 | 73.33 | capping enzyme | 73.3 |
|  |  |  | KP268809 | VP5 | 1686 | 75.15 | outer capsid protein 2 | 75.1 |
|  |  |  | MF521585 | VP6 | 991 | 80.49 | helicase | 70.0 |
|  |  |  | MF521581 | NS1 | 1748 | 73.12 | tubule protein | 83.9 |
|  |  |  | MH571972 | NS2 | 1193 | 76.52 | viral inclusion body protein | 86.5 |
|  |  |  | KP268813 | NS3 | 728 | 80.49 | NS3 protein | 88.1 |
| GTMV GD | *Riboviria* | Manly virus | MK026564 | ORF1 | 9716 | 44.9 | RdRp | 65.5 |
|  |  |  |  | ORF2 |  |  | glycoprotein | 36.5 |

Abbreviations: L, large segment; M, medium segment; S, small segment; NS, no homology with NCBI virus database; RdRp, RNA-dependent RNA polymerase; NS, no significant; BDTPV1 GD-2020, Brown dog tick phlebovirus 1 GD-2020; LTV GD-2020, Lihan tick virus GD-2020; MIV GD-2020, Mivirus sp. GD-2020; WTV1 GD-2020, Wuhan tick virus 1 GD-2020; HNV GDZJ, Hepacivirus N GDZJ; HTV2 GD-2020, Wuhan tick virus 2 GD-2020; BDTPV2 GD-2020, Brown dog tick phlebovirus 2 GD-2020; GTOV, Guangdong tick orbivirus; GTQV, Guangdong tick quaranjavirus; GTMV, Guangdong tick Manly virus.

**Supplementary Table S5.** The PCR primers used in this study.

| **Virus** | **Primer** | Position (bp)^1^ | Sequence (5'→3') | **Amplicon (bp)** |
| --- | --- | --- | --- | --- |
| HNV GD |  |  |  |  |
|  | Detection |  |  |  |
|  | HNV1-F1 | 102 | AGTACGGTCCAATCGAG |  |
|  | HNV1-R1 | 1597 | CAAATAGGATCACGGTCCAT | 1495 |
|  | HNV1-F2 | 869 | TACCGTGTTCCTTAGCGTCAA |  |
|  | HNV1-R2 | 1572 | ATCACGGTCCATGAGGCT | 703 |
|  | Amplification |  |  |  |
|  | HNV-5’-GSP-R1 | 1218 | AACCACACGACAGCGATGAGGAA |  |
|  | UPM^2^ |  |  | 1281 |
|  | HNV-5’-GSP-R2 | 866 | TTGACGCTAAGGAACACGGTAACG |  |
|  | UPM short^3^ |  | CTAATACGACTCACTATAGGGC | 866 |
|  | HNV-1-F1 | 102 | AGTACGGTCCAATCGAG |  |
|  | HNV-1-R1 | 974 | GCCGCACTGGTCATTGATG | 872 |
|  | HNV-1-F2 | 103 | GTACGGTCCAATCGAGAA |  |
|  | HNV-1-R2 | 901 | CTACGCACGACCATCATT | 798 |
|  | HNV-2-F1 | 898 | GTTCTACGCACGACCATCATT |  |
|  | HNV-2-R1 | 4540 | AAGGAAGTGGAGTGTATGC | 3642 |
|  | HNV-2-F2 | 901 | CTACGCACGACCATCATT |  |
|  | HNV-2-R2 | 4535 | TGGAGTGTATGCGAGCC | 3634 |
|  | HNV-3-F1 | 4157 | CAGCGTCACAGACTGTAATCT |  |
|  | HNV-3-R1 | 6040 | ATCCATGCGGTACTCCACAA | 1883 |
|  | HNV-3-F2 | 4183 | CTATTGAAGACGTGGAGG |  |
|  | HNV-3-R2 | 5971 | TGGCAACACCAGATGTAG | 1788 |
|  | HNV-4-F1 | 5958 | CCTGTCAACAATACTACA |  |
|  | HNV-4-R1 | 8575 | GCCAGAATAGATACAGC | 2617 |
|  | HNV-4-F1 | 5958 | CCTGTCAACAATACTACA |  |
|  | HNV-4-R2 | 8033 | TCACCTTCCGCTGAGCAA | 2075 |
|  | HNV-3’-GSP-F1 | 7354 | TTGAGACACGAGTGGCAGAGAAGA |  |
|  | UPM |  |  | 1454 |
|  | HNV-3’-GSP-F2 | 7604 | CGCACGAATCTGGAACCTACACAA |  |
|  | UPM short |  | CTAATACGACTCACTATAGGGC | 1204 |
| BDTPV1 GD |  |  |  |  |
|  | Detection |  |  |  |
|  | BDTPV1-L-F1 | 878 | CCGACCACTTGCTTGAATCA |  |
|  | BDTPV1-L-R1 | 1416 | GAGGAAGGTGTCAATGTTGCTA | 538 |
|  | BDTPV1-L-F2 | 927 | CAACTACCAAGTGACCTGTGAT |  |
|  | BDTPV1-L-R2 | 1228 | GTGTGCTCCTCATCCAGACT | 301 |
|  | BDTPV1-S-F1 | 154 | GCCTCCAAGTCCGATAAGC |  |
|  | BDTPV1-S-R1 | 628 | GTCAGGAAGACAACGCACAT | 474 |
|  | BDTPV1-S-F2 | 348 | CGCCACCATGCCTAAGGAT |  |
|  | BDTPV1-S-R2 | 619 | CAGGAAGACAACGCACATGGTGGCAAT | 271 |
|  | Amplification |  |  |  |
|  | BDTPV1-L-5’-GSP-R1 | 1061 | CTTAGAGCACTATCCAGGTCCACGGTTG |  |
|  | UPM |  |  | 1061 |
|  | BDTPV1-L-5’-GSP-R2 | 197 | AGGACCCAGCACCTTCCCTAATCTCAG |  |
|  | UPM short |  | CTAATACGACTCACTATAGGGC | 197 |
|  | BDTPV1-L-3’-GSP-F1 | 5675 | CGTCAGAGGAGGCTGCTGCATCATTG |  |
|  | UPM |  |  | 939 |
|  | BDTPV1-L-3’-GSP-F2 | 5856 | GATCTTGCAGGACTCCACCAGAACTTGT |  |
|  | UPM short |  | CTAATACGACTCACTATAGGGC | 758 |
|  | BDTPV1-S-5’-GSP-R1 | 628 | GTCAGGAAGACAACGCACAT |  |
|  | UPM |  |  | 628 |
|  | BDTPV1-S-5’-GSP-R2 | 619 | CAGGAAGACAACGCACATGGTGGCAAT |  |
|  | UPM short |  | CTAATACGACTCACTATAGGGC | 619 |
|  | BDTPV1-S-3’-GSP-F1 | 957 | CCACGCAGTCTACTTGCTTGAGTTCTCC |  |
|  | UPM |  |  | 465 |
|  | BDTPV1-S-3’-GSP-F2 | 1025 | GCCGACCTGAAGCTGTCAAGGATGG |  |
|  | UPM short |  | CTAATACGACTCACTATAGGGC | 397 |
| BDTPV2 GD |  |  |  |  |
|  | Detection |  |  |  |
|  | BDTPV2-L-F1 | 2337 | TGAAGAACAAGGACGAACCATC | 2241 |
|  | BDTPV2-L-R1 | 4578 | AGTCTTCCGTCTCGTGAACA |
|  | BDTPV2-L-F2 | 2561 | ACCATCCACATTGACATTCTCA | 1063 |
|  | BDTPV2-L-R2 | 3624 | TCTGAATTGAACTCCACCACAT |
|  | BDTPV2-S-F1 | 399 | GAAGAGCAAGACCAGCAACACCAT | 1484 |
|  | BDTPV2-S-R1 | 1883 | CACACCTCGGATGGTAAGAGA |
|  | BDTPV2-S-F2 | 962 | CAGAGACCACCAGAGGACATCACA | 351 |
|  | BDTPV2-S-R2 | 1313 | ACCGACACCAGGAGAGCAACATT |
|  | Amplification |  |  |  |
|  | BDTPV2-L-5’-GSP-R1 | 959 | CTGGCTTGTTGAAGTTCCTCCTGTATGC |  |
|  | UPM |  |  | 959 |
|  | BDTPV2-L-5’-GSP-R2 | 433 | TGGCTCTGTAGTCTGCTCGTCTG |  |
|  | UPM short |  | CTAATACGACTCACTATAGGGC | 433 |
|  | BDTPV2-L-3’-GSP-F1 | 5596 | GTCCAACATCTACCTCACGCACAA |  |
|  | UPM |  |  | 937 |
|  | BDTPV2-L-3’-GSP-F2 | 6068 | ATGGAGGATGATGGAGAGGAGGAG |  |
|  | UPM short |  | CTAATACGACTCACTATAGGGC | 465 |
|  | BDTPV2-S-5’-GSP-R1 | 764 | ATCTTGGCAGCGGTCTTGATGG |  |
|  | UPM |  |  | 764 |
|  | BDTPV2-S-5’-GSP-R2 | 513 | CTCCTTCGTTGCCACCTGCCTGTC |  |
|  | UPM short |  | CTAATACGACTCACTATAGGGC | 513 |
|  | BDTPV2-S-3’-GSP-F1 | 399 | GAAGAGCAAGACCAGCAACACCAT |  |
|  | UPM |  |  | 1696 |
|  | BDTPV2-S-3’-GSP-F2 | 962 | CAGAGACCACCAGAGGACATCACA |  |
|  | UPM short |  | CTAATACGACTCACTATAGGGC | 1133 |
| LTV GD |  |  |  |  |
|  | Detection |  |  |  |
|  | LTV-L-F1 | 2041 | GGACCGAAGAGATTATCACA | 825 |
|  | LTV-L-R1 | 2866 | TCTCCAGTGCCAGTTGTA |  |
|  | LTV-L-F2 | 2139 | CCAGATGTTGCTAGGACTC | 427 |
|  | LTV-L-R2 | 2566 | TGCGTTCAGAATGTCTATGT |  |
|  | LTV-S-F1 | 424 | GAAATCCACCGACAAACG | 1088 |
|  | LTV-S-R1 | 1512 | CATGTTGTGCCCAGTGTA |  |
|  | LTV-S-F2 | 510 | ACAGGAGGTCCAAGTGGCGGAACAG |  |
|  | LTV-S-R2 | 1261 | GGCAGACAGGACTCCAGCACCACAG | 751 |
|  | Amplification |  |  |  |
|  | LTV-L-5’-GSP-R1 | 657 | CACGGAGGTCAGGAAGCATGGATCTACT |  |
|  | UPM |  |  | 657 |
|  | LTV-L-5’-GSP-R2 | 612 | AATCTACCGCAGTGCTGAACCTGAGACA |  |
|  | UPM short |  | CTAATACGACTCACTATAGGGC | 612 |
|  | LTV-L-3’-GSP-F1 | 6020 | GCAAGCCAGAGCACTCCTT |  |
|  | UPM |  |  | 497 |
|  | LTV-L-3’-GSP-F2 | 6340 | AGACAAGAGCCAAGGAACTACTA |  |
|  | UPM short |  | CTAATACGACTCACTATAGGGC | 177 |
|  | LTV-S-5’-GSP-R1 | 556 | GGCTCATCATCCTCGTCGTCACTGTTC |  |
|  | UPM |  |  | 556 |
|  | LTV-S-5’-GSP-R2 | 307 | CCACCATGCTCGTCATCACCTGGATTGT |  |
|  | UPM short |  | CTAATACGACTCACTATAGGGC | 307 |
|  | LTV-S-3’-GSP-F1 | 1038 | GCCACAATTCTAGCCGCCCACCACTCTG |  |
|  | UPM |  |  | 724 |
|  | LTV-S-3’-GSP-F2 | 1132 | TGACGCAGGCAGTGGCAGCAGCAAT |  |
|  | UPM short |  | CTAATACGACTCACTATAGGGC | 630 |
| MIV GD |  |  |  |  |
|  | Detection |  |  |  |
|  | MIV-F1 | 8311 | ATGGATGGTGGTGGACTT | 610 |
|  | MIV-R1 | 8921 | TCAAGATAGGCAACGATAGG |  |
|  | MIV-F1 | 8311 | ATGGATGGTGGTGGACTT | 271 |
|  | MIV-R2 | 8582 | AGACCTGGATTCACTCACA |  |
|  | Amplification |  |  |  |
|  | MIV-5’-GSP-R1 | 1006 | CTCCGTGAGCAGGCGTTCAAGTG |  |
|  | UPM |  |  | 1006 |
|  | MIV-5’-GSP-R2 | 1001 | CGTGAGCAGGCGTTCAAGTGGTGAG |  |
|  | UPM short |  | CTAATACGACTCACTATAGGGC | 1001 |
|  | MIV-3’-GSP-F1 | 10730 | ATGATGGAACGATACGATGTCTG |  |
|  | UPM |  |  | 541 |
|  | MIV-3’-GSP-F2 | 10804 | CTCCGAGACCCGCCTCACCCTCACTAT |  |
|  | UPM short |  | CTAATACGACTCACTATAGGGC | 467 |
| WTV1 GD |  |  |  |  |
|  | Detection |  |  |  |
|  | WTV1-F1 | 4470 | GCGACAGAGAAGTTGACA |  |
|  | WTV1-R1 | 6150 | GAGCAACTGTCAAGGAACT | 1680 |
|  | WTV1-F2 | 5035 | TTCCTCTCCTTCCTTGCTA |  |
|  | WTV1-R2 | 5524 | CCTTCCGTTGATGGTTGT | 489 |
|  | Amplification |  |  |  |
|  | WTV1-5’-GSP-R1 | 935 | AGGCTGAAGTCGGCACAGTAGTGGTAAT |  |
|  | UPM |  |  | 935 |
|  | WTV1-5’-GSP-R2 | 555 | TCGGCTTCTGACGGTCTTGATGTACTCT |  |
|  | UPM short |  | CTAATACGACTCACTATAGGGC | 555 |
|  | WTV1-3’-GSP-F1 | 9468 | GGAGTGGCGTCTGATCTCGCATTCA |  |
|  | UPM |  |  | 1930 |
|  | WTV1-3’-GSP-F2 | 9602 | CATTGCTCCTCCTTCTGACCAACGACTT |  |
|  | UPM short |  | CTAATACGACTCACTATAGGGC | 1796 |
| WTV2 GD |  |  |  |  |
|  | Detection |  |  |  |
|  | WTV2-F1 | 2212 | AACACTCCACAACACTACC |  |
|  | WTV2-R1 | 3033 | CACCACCTTCCTTCAATCA | 821 |
|  | WTV2-F2 | 2310 | TATGAACGGCGGAGATAAG |  |
|  | WTV2-R2 | 2965 | GCAGAACCAGAAGAAGGAA | 655 |
|  | Amplification |  |  |  |
|  | WTV1-5’-GSP-R1 | 1337 | CCAGACCACCAGAACCATCACATTG |  |
|  | UPM |  |  | 1337 |
|  | WTV1-5’-GSP-R2 | 1076 | CATCGTAAACCTTAGGCTCGCCATC |  |
|  | UPM short |  | CTAATACGACTCACTATAGGGC | 1076 |
|  | WTV1-3’-GSP-F1 | 10046 | CAATGCGAATGGCTCCGTACTGTT |  |
|  | UPM |  |  | 1695 |
|  | WTV1-3’-GSP-F2 | 10063 | CGCATAGACCAGCACGGATAAGAT |  |
|  | UPM short |  | CTAATACGACTCACTATAGGGC | 1678 |
| GTQV |  |  |  |  |
|  | Detection |  |  |  |
|  | GTQV-F1 | 5' | AGCAGAGGCAGCWYGT | 930 |
|  | GTQV-R1 | 3' | AGTAGAGACAAGCWYGTTT |  |
|  | Amplification |  |  |  |
|  | GTQV-PB1-5’-GSP-R1 | 1292 | TGCTGCTCGGCTCTCATGTACCATTCCT |  |
|  | UPM |  |  | 1292 |
|  | GTQV-PB1-5’-GSP-R2 | 772 | TCAAGTACGCACAGAAGCTCACGGC |  |
|  | UPM short |  | CTAATACGACTCACTATAGGGC | 772 |
|  | GTQV-PB1-F1 | 740 | ATGAGCAGGTCTACAATGAA |  |
|  | GTQV-PB1-R1 | 1332 | GTATGGAGCCTTGAGGTAC | 592 |
|  | GTQV-PB1-F2 | 912 | CGGTAGTGTCATAGGAGTC |  |
|  | GTQV-PB1-R2 | 1273 | TGGTCTTGTCGTTCATCAT | 361 |
|  | GTQV-PB1-3’-GSP-F1 | 1705 | TCTCAGACAGCCACTTCACTCAGGACCG |  |
|  | UPM |  |  | 718 |
|  | GTQV-PB1-3’-GSP-F2 | 1879 | TGGAATTGGTCTACCTGTCACTTGCCTG |  |
|  | UPM short |  | CTAATACGACTCACTATAGGGC | 544 |
|  | GTQV-PB2-5’-GSP-R1 | 521 | CAGTGGCAGCCTGGTCTGGACTCTTCTT |  |
|  | UPM |  |  | 521 |
|  | GTQV-PB2-5’-GSP-R2 | 517 | GCAGCCTGGTCTGGACTCTTCTTCTTG |  |
|  | UPM short |  | CTAATACGACTCACTATAGGGC | 517 |
|  | GTQV-PB2--F1 | 875 | GGCTGACCTGAATCTACTT |  |
|  | GTQV-PB2--R1 | 1447 | TCTACTGGTAAGGCTGGAT | 572 |
|  | GTQV-PB2--F2 | 1096 | CAACGGAAGTAAGAGTCAAC |  |
|  | GTQV-PB2--R2 | 1396 | GAATTGTGTCCAGTTCTGAG | 300 |
|  | GTQV-PB2-3’-GS-F1 | 1930 | CACTTGCCACTGTGAACTCCGCCATCTT |  |
|  | UPM |  |  | 459 |
|  | GTQV-PB2-3’-GSP-F2 | 1932 | CTTGCCACTGTGAACTCCGCCATCT |  |
|  | UPM short |  | CTAATACGACTCACTATAGGGC | 457 |
|  | GTQV-NP-5’-GSP-R1 | 1312 | TTGTGGTGGCTGCTTCCTTCATCAGAGT |  |
|  | UPM |  |  | 1312 |
|  | GTQV-NP-5’-GSP-R2 | 695 | CTGGCACAACGGTCCAAGTGACTGAG |  |
|  | UPM short |  | CTAATACGACTCACTATAGGGC | 695 |
|  | GTQV-NP--F1 | 556 | GGCATCGTAACCAGTAAGA |  |
|  | GTQV-NP--R1 | 1076 | GTCGTTGACATTCATTGAGA | 520 |
|  | GTQV-NP--F2 | 676 | TTCGTGAAGAGTGGCATAG |  |
|  | GTQV-NP--R2 | 946 | TCTCGGACTGTCAAGTAATG | 270 |
|  | GTQV-NP-3’-GSP-F1 | 695 | CTCAGTCACTTGGACCGTTGTGCCAG |  |
|  | UPM |  |  | 1050 |
|  | GTQV-NP-3’-GSP-F2 | 1312 | ACTCTGATGAAGGAAGCAGCCACCACAA |  |
|  | UPM short |  | CTAATACGACTCACTATAGGGC | 433 |
|  | GTQV-HA-5’-GSP-R1 | 1482 | AGCGGCTCCAGAAGGTCCAGAAGG |  |
|  | UPM |  |  | 1482 |
|  | GTQV-HA-5’-GSP-R2 | 734 | CTCTCAGTCGGTCATCTTCAGGCAGTTG |  |
|  | UPM short |  | CTAATACGACTCACTATAGGGC | 734 |
|  | GTQV-HA--F1 | 459 | GCAACAACGAGGAATGGA |  |
|  | GTQV-HA--R1 | 1269 | ATGTGTCAGGTTCAAGGTAG | 810 |
|  | GTQV-HA--F2 | 743 | GAAGATGACCGACTGAGAG |  |
|  | GTQV-HA--R2 | 1134 | GATAACATGGACAGAGATGGA | 391 |
|  | GTQV-HA-3’-GSP-F1 | 224 | AGTAGGTACACGGCGTACTGCTATGAGG |  |
|  | UPM |  |  | 1420 |
|  | GTQV-HA-3’-GSP-F2 | 459 | CAACTGCCTGAAGATGACCGACTGAGAG |  |
|  | UPM short |  | CTAATACGACTCACTATAGGGC | 1185 |
|  | GTQV-PA-5’-GSP-R1 | 1410 | ATCCTGTCCGTGGTGGTGGCTGCTTCA |  |
|  | UPM |  |  | 1410 |
|  | GTQV-PA-5’-GSP-R2 | 1178 | GCTCGTGCTGCCTGCCAGTTGAC |  |
|  | UPM short |  | CTAATACGACTCACTATAGGGC | 1178 |
|  | GTQV-PA-3’-GSP-F1 | 1410 | TGAAGCAGCCACCACCACGGACAGGAT |  |
|  | UPM |  |  | 970 |
|  | GTQV-PA-3’-GSP-F2 | 1816 | GCGTGAAGCAACCGACAAGGTCAAC |  |
|  | UPM short |  | CTAATACGACTCACTATAGGGC | 564 |
|  | GTQV-M-F1 | 5' | AGCAGAGGCAGCWYGT |  |
|  | GTQV-M-R1 | 3' | AGTAGAGACAAGCWYGTTT | 930 |
| GTMV |  |  |  |  |
|  | Amplification |  |  |  |
|  | GTMV F1 | 5673 | TTGGAAGGATTACGACAGAA |  |
|  | GTMV R1 | 9427 | TGGAATTGGAGAGCAGGAT | 3754 |
|  | GTMV F2 | 6678 | TTCTACGCAGCAACCTTC |  |
|  | GTMV R2 | 9173 | TGTCAAGGCATAGGATAACG | 2495 |

Abbreviations: BDTPV1 GD, Brown dog tick phlebovirus 1 GD; LTV GD, Lihan tick virus GD; MIV GD, Mivirus sp. GD; WTV1 GD, Wuhan tick virus 1 GD; HNV GD, Hepacivirus N GD; HTV2 GD, Wuhan tick virus 2 GD; BDTPV2 GD, Brown dog tick phlebovirus 2 GD; GTOV, Guangdong tick orbivirus; GTQV, Guangdong tick quaranjavirus; GTMV, Guangdong tick Manly virus.

1 The position of primers referred to the complete sequences obtained from RNA-seq in this study.

2 UPM: Universal Primer A Mix provided by 5’/3’ RACE kits (TaKaRa, Japan).

3 UPM short: Universal Primer short provided by 5’/3’ RACE kits (TaKaRa, Japan).

**Supplementary Table S6.** GenBank accession number and abbreviation of virus in the study

| Virus name | Virus abbreviation | Accession number |
| --- | --- | --- |
| Brown dog tick phlebovirus 1 strain GD-JY02-1 | BDTPV1 GD-JY02-1 | Segment L (OM240632), Segment S (OM240633) |
| Brown dog tick phlebovirus 1 strain GD-JY01 | BDTPV1 GD-JY01 | Segment L (OM326742), Segment S (OM326743) |
| Brown dog tick phlebovirus 1 strain GD-JY02-2 | BDTPV1 GD-JY02-2 | Segment L (OM326744), Segment S (OM326745) |
| Brown dog tick phlebovirus 1 strain GD-JY02-3 | BDTPV1 GD-JY02-3 | Segment L (OM326746), Segment S (OM326747) |
| Brown dog tick phlebovirus 1 strain GD-ZJ0103 | BDTPV1 GD-ZJ0103 | Segment L (OM326748), Segment S (OM326749) |
| Brown dog tick phlebovirus 2 strain GD-JY02-1 | BDTPV2 GD-JY02-1 | Segment L (OM263650), Segment S (OM263651) |
| Brown dog tick phlebovirus 2 strain GD-JY01 | BDTPV2 GD-JY01 | Segment L (OM326751), Segment S (OM326753) |
| Brown dog tick phlebovirus 2 strain GD-JY02-3 | BDTPV2 GD-JY02-3 | Segment L (OM326752), Segment S (OM326754) |
| Lihan tick virus strain GD-ZJ02 | LTV GD-ZJ02 | Segment L (OM240634), Segment S (OM240635) |
| Lihan tick virus strain GD-JY02-3 | LTV GD-JY02-3 | Segment L (OM326750) |
| Mivirus sp. strain GD | MIV GD | OM240631 |
| Wuhan tick virus 2 strain GD-JY02-3 | WTV2 GD-JY02-3 | OM264150 |
| Wuhan tick virus 2 strain GD-ZJ0103 | WTV2 GD-ZJ0103 | OM326755 |
| Wuhan tick virus 1 strain GD-JY02-3 | WTV1 GD-JY02-3 | OM264163 |
| Wuhan tick virus 1 strain GD-ZJ0103 | WTV1 GD-ZJ0103 | OM264160 |
| Wuhan tick virus 1 strain GD-ZJ02 | WTV1 GD-ZJ02 | OM264161 |
| Wuhan tick virus 1 strain GD-JY02-1 | WTV1 GD-JY02-1 | OM264162 |
| Hepacivirus N isolate GDZJ | HNV GDZJ | MZ221927 |
| Hepacivirus N isolate GDZJ02-02 | HNV GDZJ02-02 | MZ540979 |
| Hepacivirus N isolate GDZJ02-03 | HNV GDZJ02-03 | MZ540980 |
| Guangdong tick quaranjavirus | GTQV | PB2/PA/PB1/HA/NP/M: OM273643-OM273648 |
| Guangdong tick orbivirus^*^ | GTOV | VP1/VP2/VP3/VP4/VP5/VP6/NS1/NS2/NS3: OM264151-OM264159 |
| Guangdong tick manly virus | GTMV | OM264164 |

^*^Only partial genome obtained in this study.

**Supplementary Table S7.** The mean depth of viral sequencing in each tick pool^*^

| **Virus name** | **GDJY/01** | **GDJY/02/1** | **GDJY/02/2** | **GDJY/02/3** | **GDZJ/02** | **GDZJ/0103** |
| --- | --- | --- | --- | --- | --- | --- |
| BDTPV1 GD L | 68.83 | 419.97 | 65.24 | 8.96 |  | 96.04 |
| BDTPV1 GD- S | 449.09 | 1976.53 | 268.75 | 29.76 |  | 150.93 |
| BDTPV2 GD L | 236.53 | 591.64 | 210.75 | 62.53 |  | 26.61 |
| BDTPV2 GD S | 378.47 | 513.68 | 318.29 | 74.93 |  | 60.57 |
| LTV GD L |  |  |  | 26.07 | 14.03 | 5.14 |
| LTV GD S |  |  |  | 28.60 | 16.74 | 3.70 |
| MIV GD | 246.66 | 230.44 | 10.31 |  |  |  |
| WTV2 GD |  | 109.86 |  | 129.46 |  | 8.30 |
| WTV1 GD |  | 12.57 |  | 44.93 | 29.21 | 30.88 |
| HNV GDZJ |  |  |  |  | 3.82 |  |
| GTQV PB2 | 24.88 | 269.02 | 64.92 | 3.68 |  | 3.44 |
| GTQV PA | 50.10 | 486.60 | 81.76 | 3.02 |  | 6.21 |
| GTQV PB1 | 69.72 | 356.88 | 72.64 | 3.54 |  | 5.08 |
| GTQV HA | 155.41 | 713.17 | 295.75 | 4.86 |  | 6.67 |
| GTQV NP | 35.80 | 214.45 | 76.92 | 3.63 |  | 3.11 |
| GTQV M | 298.36 | 1088.18 | 610.03 | 5.18 |  | 9.24 |
| GTOV VP1 |  |  |  |  | 3.85 |  |
| GTOV VP2 |  |  |  |  | 4.20 |  |
| GTOV VP3 |  |  |  |  | 3.05 |  |
| GTOV VP4 |  |  |  |  | 2.27 |  |
| GTOV VP5 |  |  |  |  | 3.67 |  |
| GTOV VP6 |  |  |  |  | 9.79 |  |
| GTOV NS1 |  |  |  |  | 2.69 |  |
| GTOV NS2 |  |  |  |  | 4.91 |  |
| GTOV NS3 |  |  |  |  | 14.51 |  |
| GTMV |  | 8.02 |  | 3.65 |  |  |

^*^BDTPV1 GD, Brown dog tick phlebovirus 1 Guangdong strain; LTV GD, Lihan tick virus Guangdong strain; MIV GD, Mivirus Guangdong strain; WTV1 GD, Wuhan tick virus 1 Guangdong strain; HNV GD, Hepacivirus N Guangdong strain; HTV2 GD, Wuhan tick virus 2 Guangdong strain; BDTPV2 GD, Brown dog tick phlebovirus 2 Guangdong strain; GTOV, Guangdong tick orbivirus; GTQV, Guangdong tick quaranjavirus; GTMV, Guangdong tick Manly virus; L, large segment; S, small segment.

**Supplementary Table S8. The sequence identity within the LTV, BDTPV1 and BDTPV2^*^**

|  | Strains | 1 | 2 | 3 | 4 | 5 | 6 | 7 | 8 | 9 | 10 | 11 | 12 | 13 | 14 | 15 | 16 | 17 | 18 | 19 | 20 | 21 | 22 |
| --- | --- | --- | --- | --- | --- | --- | --- | --- | --- | --- | --- | --- | --- | --- | --- | --- | --- | --- | --- | --- | --- | --- | --- |
| 1 | OM240635/LTV/China |  | 94.5 | 58.4 | 58.4 | 58.4 | 58.5 | 58.8 | 61.2 | 61.2 | 61 | 61.5 | 90.8 | 95.2 | 95.8 | 96.4 | 96.1 | 95.8 | 95.8 | 95.7 | 95.7 | 58.7 | 60.7 |
| 2 | OM326750/LTV/China | 98.6 |  | 58.4 | 58.4 | 58.4 | 58.5 | 58.8 | 60.8 | 60.9 | 60.6 | 61.2 | 90.5 | 93.2 | 93.8 | 94.4 | 94.2 | 93.8 | 94.3 | 94.1 | 94.2 | 58.6 | 60.5 |
| 3 | OM326742/BDTPV1/China | 52.5 | 52.2 |  | 99.9 | 100 | 99 | 98 | 62.8 | 62.8 | 62.6 | 63.4 | 58.4 | 57.8 | 57.9 | 58.2 | 58.1 | 58 | 58.5 | 58.2 | 58.3 | 95.8 | 61.9 |
| 4 | OM326744/BDTPV1/China | 52.5 | 52.2 | 100 |  | 100 | 99 | 97.9 | 62.8 | 62.8 | 62.6 | 63.4 | 58.4 | 57.8 | 57.9 | 58.2 | 58.1 | 58 | 58.5 | 58.2 | 58.2 | 95.8 | 61.9 |
| 5 | OM240632/BDTPV1/China | 52.5 | 52.2 | 100 | 100 |  | 99 | 97.9 | 62.8 | 62.8 | 62.6 | 63.4 | 58.4 | 57.8 | 57.9 | 58.2 | 58.1 | 58 | 58.5 | 58.2 | 58.2 | 95.8 | 61.9 |
| 6 | OM326748/BDTPV1/China | 52.5 | 52.2 | 99.7 | 99.8 | 99.8 |  | 97.7 | 62.9 | 62.9 | 62.7 | 63.5 | 58.4 | 57.9 | 58 | 58.4 | 58.3 | 58.2 | 58.6 | 58.4 | 58.4 | 95.9 | 62 |
| 7 | OM326746/BDTPV1/China | 52.4 | 52.1 | 99.6 | 99.5 | 99.5 | 99.4 |  | 63.1 | 63.1 | 62.9 | 63.6 | 58.8 | 58.8 | 58.9 | 58.6 | 59 | 58.9 | 59 | 58.9 | 59.2 | 94.5 | 62.6 |
| 8 | OM263650/BDTPV2/China | 56 | 55.9 | 57.6 | 57.6 | 57.6 | 57.7 | 57.8 |  | 99.9 | 99.3 | 85 | 61.3 | 60.3 | 60.6 | 60.9 | 60.8 | 60.8 | 60.7 | 60.4 | 60.4 | 63.3 | 78.8 |
| 9 | OM326751/BDTPV2/China | 56 | 55.9 | 57.6 | 57.6 | 57.6 | 57.7 | 57.8 | 100 |  | 99.3 | 85 | 61.3 | 60.3 | 60.6 | 61 | 60.8 | 60.8 | 60.8 | 60.4 | 60.4 | 63.3 | 78.8 |
| 10 | OM326752/BDTPV2/China | 55.9 | 55.8 | 57.6 | 57.6 | 57.6 | 57.6 | 57.7 | 99.7 | 99.7 |  | 84.8 | 61 | 60.1 | 60.3 | 60.7 | 60.6 | 60.6 | 60.5 | 60.2 | 60.2 | 63.1 | 78.6 |
| 11 | MN025508/BDTPV2/Trinidad and Tobago | 55.7 | 55.6 | 58 | 58 | 58 | 58 | 58.1 | 96.7 | 96.8 | 96.6 |  | 61.3 | 60.9 | 61.1 | 61.5 | 61.4 | 61.3 | 61.3 | 61.1 | 61 | 63.5 | 79.1 |
| 12 | MN095537/LTV/Thailand | 97.6 | 97.5 | 52.2 | 52.2 | 52.2 | 52.2 | 52.2 | 55.6 | 55.6 | 55.5 | 55.2 |  | 89.7 | 90.1 | 90.7 | 90.5 | 90.2 | 90.8 | 90.8 | 90.6 | 58.6 | 61.1 |
| 13 | MH155912/LTV/Brazil | 98.6 | 98 | 52.3 | 52.3 | 52.3 | 52.3 | 52.2 | 56 | 56 | 55.9 | 55.8 | 96.9 |  | 98.3 | 98.1 | 98.4 | 98.1 | 94.5 | 95.1 | 95.1 | 58.1 | 60.9 |
| 14 | MH155914/LTV/Brazil | 99 | 98.6 | 52.5 | 52.5 | 52.5 | 52.5 | 52.4 | 56 | 56 | 55.9 | 55.7 | 97.4 | 99.2 |  | 98.8 | 99.1 | 98.7 | 95.2 | 95.6 | 95.7 | 58.3 | 60.7 |
| 15 | MN599996/LTV/Guadeloupe | 99.1 | 98.6 | 52.5 | 52.5 | 52.5 | 52.5 | 52.4 | 56 | 56 | 55.9 | 55.7 | 97.6 | 99.3 | 99.8 |  | 99.3 | 98.8 | 95.6 | 95.5 | 95.5 | 58.6 | 60.8 |
| 16 | MK683459/LTV/Colombia | 99.2 | 98.6 | 52.4 | 52.4 | 52.4 | 52.4 | 52.3 | 56 | 56 | 55.9 | 55.7 | 97.5 | 99.2 | 99.8 | 99.9 |  | 99.4 | 95.5 | 95.7 | 95.8 | 58.5 | 60.9 |
| 17 | MN025510/LTV/Trinidad and Tobago | 98.9 | 98.4 | 52.5 | 52.5 | 52.5 | 52.5 | 52.4 | 56.1 | 56.1 | 56 | 55.8 | 97.3 | 99.1 | 99.6 | 99.7 | 99.8 |  | 95.1 | 95.4 | 95.6 | 58.4 | 60.8 |
| 18 | NC055423/LTV/China | 98.9 | 98.3 | 52.4 | 52.4 | 52.4 | 52.4 | 52.3 | 55.7 | 55.7 | 55.6 | 55.4 | 97.7 | 98.3 | 98.7 | 98.8 | 98.8 | 98.6 |  | 98.6 | 98.6 | 58.8 | 60.6 |
| 19 | MW721905/LTV/China | 99.3 | 98.5 | 52.5 | 52.5 | 52.5 | 52.5 | 52.4 | 55.9 | 55.9 | 55.8 | 55.6 | 98 | 98.5 | 99 | 99.1 | 99.1 | 98.9 | 99.6 |  | 99 | 58.6 | 61 |
| 20 | MW721911/LTV/China | 99.1 | 98.5 | 52.5 | 52.5 | 52.5 | 52.5 | 52.4 | 55.8 | 55.8 | 55.7 | 55.6 | 97.8 | 98.3 | 98.9 | 98.9 | 98.9 | 98.7 | 99.4 | 99.7 |  | 58.6 | 60.9 |
| 21 | MN025506/BDTPV1/Trinidad and Tobago | 52.5 | 52.2 | 99.3 | 99.3 | 99.3 | 99.4 | 98.9 | 57.8 | 57.8 | 57.7 | 58.1 | 52.2 | 52.3 | 52.5 | 52.5 | 52.4 | 52.5 | 52.4 | 52.5 | 52.5 |  | 62.2 |
| 22 | MW561136/BDTPV2/Romania | 55.1 | 55.1 | 56.7 | 56.7 | 56.7 | 56.7 | 56.7 | 88.6 | 88.7 | 88.5 | 89 | 54.6 | 55.1 | 55.1 | 55.1 | 55.1 | 55.1 | 54.8 | 55 | 54.9 | 56.8 |  |

Percent similarity at the complete nucleotide of segment L (upper triangle) and amino acid secquence of RdRp (lower triangle) levels calculated using ClustalW method implemented in MegAlign.

* LTV, Lihan tick virus; BDTPV1, Brown dog tick phlebovirus 1; BDTPV2, Brown dog tick phlebovirus 2.

**Supplementary Table S9. The sequence identity within the MIV and WTV2^*^**

|  |  | 1 | 2 | 3 | 4 | 5 | 6 | 7 | 8 | 9 | 10 | 11 | 12 | 13 | 14 | 15 | 16 | 17 | 18 | 19 |
| --- | --- | --- | --- | --- | --- | --- | --- | --- | --- | --- | --- | --- | --- | --- | --- | --- | --- | --- | --- | --- |
| 1 | OM240631/MIV/China |  | 43.3 | 43.3 | 43.2 | 43.3 | 88.1 | 96.7 | 42.7 | 43.2 | 43.1 | 43.1 | 43.1 | 42.6 | 43.2 | 42.6 | 41.7 | 40.7 | 38.8 | 43.2 |
| 2 | OM264150/WTV2/China | 52.9 |  | 98.9 | 96.9 | 97.6 | 42.5 | 42.4 | 82.1 | 92.5 | 92.5 | 92.4 | 92.5 | 91.1 | 92.4 | 91.1 | 89.6 | 85.1 | 82.6 | 92.3 |
| 3 | OM326755/WTV2/China | 52.9 | 99.9 |  | 96.9 | 97.6 | 42.5 | 42.4 | 82.1 | 92.4 | 92.4 | 92.4 | 92.4 | 91.1 | 92.3 | 91.1 | 89.6 | 85.1 | 82.5 | 92.3 |
| 4 | MZ965027/WTV2/China | 14.7 | 15.7 | 15.7 |  | 99.3 | 43.8 | 43 | 81.2 | 91.7 | 91.7 | 91.5 | 91.6 | 92.4 | 91.6 | 92.4 | 89.8 | 86.4 | 83.8 | 91.4 |
| 5 | MW721953/WTV2/China | 52.8 | 99.6 | 99.5 | 15.7 |  | 43.1 | 42.9 | 81.8 | 92.4 | 92.4 | 92.2 | 92.3 | 91.7 | 92.3 | 91.7 | 89.5 | 85.7 | 83.1 | 92.1 |
| 6 | MN095545/Changping mivirus/Thailand | 99 | 52.6 | 52.6 | 14.5 | 52.5 |  | 88.6 | 42 | 42.4 | 42.3 | 42.4 | 42.4 | 43.3 | 42.4 | 43.3 | 41.6 | 44.1 | 45.4 | 42.3 |
| 7 | MN025520/Mivirus sp./TrinidadandTobago | 99.5 | 52.9 | 52.9 | 14.7 | 52.8 | 99.1 |  | 41.9 | 42.3 | 42.2 | 42.2 | 42.2 | 42.4 | 42.3 | 42.4 | 40.7 | 40.7 | 38.7 | 42.2 |
| 8 | MN095546/Wuhan mivirus/Thailand | 53 | 94.5 | 94.4 | 15.2 | 94.5 | 52.6 | 52.9 |  | 82.5 | 82.5 | 82.5 | 82.5 | 81.2 | 82.5 | 81.3 | 80 | 76 | 73.8 | 82.4 |
| 9 | MW721951/WTV2/China | 52.8 | 98.1 | 98.1 | 15.6 | 98.2 | 52.5 | 52.8 | 94.3 |  | 99 | 94.6 | 94.9 | 93.5 | 99.1 | 93.5 | 91.9 | 87.3 | 84.8 | 95 |
| 10 | MW721943/WTV2/China | 52.9 | 98.2 | 98.1 | 15.6 | 98.3 | 52.5 | 52.9 | 94.4 | 99.9 |  | 94.6 | 94.8 | 93.4 | 98.9 | 93.4 | 91.8 | 87.2 | 84.7 | 94.9 |
| 11 | MK683460/WTV2/Colombia | 53 | 98.1 | 98 | 15.6 | 98.1 | 52.7 | 53 | 94.2 | 98.7 | 98.6 |  | 98.9 | 97.8 | 94.6 | 97.6 | 96.4 | 91.1 | 88.3 | 94.4 |
| 12 | MN599999/WTV2/Guadeloupe | 53 | 98.1 | 98 | 15.5 | 98.1 | 52.6 | 53 | 94.4 | 98.7 | 98.7 | 99.8 |  | 98 | 94.8 | 98 | 96 | 91.3 | 88.7 | 94.6 |
| 13 | MH155927/WTV2/Brazil | 52.3 | 95 | 94.9 | 15.1 | 95 | 51.9 | 52.3 | 91.6 | 95.7 | 95.6 | 96.6 | 96.6 |  | 93.5 | 99.3 | 96.8 | 92.9 | 89.9 | 93.2 |
| 14 | NC028266/WTV2/China | 14.7 | 15.7 | 15.7 | 98.1 | 15.8 | 14.5 | 14.7 | 15.2 | 15.7 | 15.7 | 15.7 | 15.6 | 15.2 |  | 93.5 | 91.8 | 87.3 | 84.7 | 94.9 |
| 15 | MH155923/WTV2/Brazil | 52.3 | 94.9 | 94.9 | 15.1 | 95 | 51.9 | 52.3 | 91.6 | 95.6 | 95.5 | 96.5 | 96.5 | 99.9 | 15.2 |  | 96.3 | 92.7 | 90 | 93.2 |
| 16 | MN025521/WTV2/TrinidadandTobago | 52.2 | 94.9 | 94.9 | 15.1 | 95 | 51.9 | 52.2 | 91.5 | 95.5 | 95.5 | 96.6 | 96.5 | 99.8 | 15.2 | 99.7 |  | 91 | 87 | 91.7 |
| 17 | MH155921/WTV2/Brazil | 51.5 | 91.8 | 91.8 | 15.5 | 91.9 | 51.1 | 51.5 | 88.5 | 92.3 | 92.3 | 93.3 | 93.3 | 96.6 | 15.5 | 96.6 | 96.4 |  | 89.6 | 87 |
| 18 | MW721952/WTV2/China | 52.8 | 98 | 97.9 | 15.6 | 98.1 | 52.5 | 52.8 | 94.5 | 98.6 | 98.7 | 98.5 | 98.6 | 95.5 | 15.7 | 95.4 | 95.4 | 92.2 |  | 84.4 |
| 19 | MH155920/WTV2/Brazil | 49.4 | 89 | 89 | 15.5 | 89.1 | 49 | 49.4 | 85.8 | 89.6 | 89.5 | 90.5 | 90.5 | 93.8 | 15.5 | 93.8 | 93.6 | 92.6 | 89.5 |  |

Percent similarity at the nucleotide (upper triangle) and amino acid of RdRp (lower triangle) levels calculated using ClustalW method implemented in MegAlign.

^*^MIV, Mivirus; WTV2, Wuhan tick virus 2.

**Supplementary Table S10**. The sequence identity comparison of Wuhan tick virus 1^*^

|  | Strains | 1 | 2 | 3 | 4 | 5 | 6 | 7 | 8 | 9 | 10 | 11 | 12 | 13 | 14 | 15 | 16 | 17 | 18 | 19 | 20 | 21 | 22 | 23 | 24 |
| --- | --- | --- | --- | --- | --- | --- | --- | --- | --- | --- | --- | --- | --- | --- | --- | --- | --- | --- | --- | --- | --- | --- | --- | --- | --- |
| 1 | OM264163/WTV1/China |  | 98.7 | 95.6 | 95.4 | 95.3 | 95.1 | 95.4 | 95.5 | 95.2 | 95.6 | 95.6 | 95.5 | 95.5 | 95.5 | 95 | 95.4 | 95.2 | 95.2 | 95 | 95.5 | 95.3 | 95.3 | 89 | 73.5 |
| 2 | OM264162/WTV1/China | 99.6 |  | 95.5 | 95.4 | 95.3 | 95.1 | 95.4 | 95.4 | 95.1 | 95.5 | 95.5 | 95.4 | 95.4 | 95.4 | 94.9 | 95.3 | 95.1 | 95.1 | 94.9 | 95.4 | 95.1 | 95.2 | 89 | 73.5 |
| 3 | OM264160/WTV1/China | 99.4 | 99.2 |  | 99.2 | 95.1 | 94.9 | 95.3 | 95.4 | 95.1 | 95.5 | 95.5 | 95.4 | 95.4 | 95.3 | 94.9 | 95.3 | 95.1 | 95.1 | 94.8 | 95.4 | 95.1 | 95.2 | 89 | 73.3 |
| 4 | OM264161/WTV1/China | 99.4 | 99.2 | 99.7 |  | 95 | 94.7 | 95.2 | 95.3 | 95 | 95.4 | 95.4 | 95.3 | 95.3 | 95.2 | 94.8 | 95.2 | 95 | 95 | 94.7 | 95.2 | 95 | 95.1 | 88.9 | 73.3 |
| 5 | MW721933/WTV1/China | 99.3 | 99.1 | 99.4 | 99.4 |  | 99.3 | 98.1 | 97.6 | 97.3 | 97.7 | 97.6 | 97.6 | 97.5 | 97.5 | 96.7 | 97 | 97.3 | 97.3 | 97.1 | 97.5 | 97.3 | 97.3 | 88.6 | 73.8 |
| 6 | MZ965021/WTV1/China | 99.3 | 99.1 | 99.4 | 99.4 | 100 |  | 97.4 | 96.9 | 96.6 | 97 | 97 | 96.9 | 96.9 | 96.9 | 97.3 | 97.7 | 96.6 | 96.6 | 96.4 | 96.9 | 96.6 | 96.7 | 88.2 | 74.5 |
| 7 | MW721932/WTV1/China | 99.4 | 99.2 | 99.5 | 99.5 | 99.9 | 99.9 |  | 99 | 98.8 | 99.2 | 99.2 | 99.1 | 99.1 | 99 | 98.1 | 98.5 | 98.8 | 98.8 | 98.6 | 99 | 98.8 | 98.8 | 88.7 | 73.7 |
| 8 | MW721931/WTV1/China | 99.4 | 99.3 | 99.6 | 99.5 | 99.6 | 99.6 | 99.8 |  | 99.6 | 99.8 | 99.7 | 99.9 | 99.9 | 99.9 | 99 | 99.1 | 99.6 | 99.6 | 99.4 | 99.9 | 99.6 | 99.6 | 88.6 | 73.8 |
| 9 | MW721930/WTV1/China | 99.4 | 99.2 | 99.5 | 99.5 | 99.7 | 99.7 | 99.7 | 100 |  | 99.5 | 99.5 | 99.5 | 99.6 | 99.5 | 99.3 | 98.8 | 99.4 | 99.4 | 99.2 | 99.6 | 99.4 | 99.3 | 88.5 | 73.6 |
| 10 | MW721927/WTV1/China | 99.4 | 99.3 | 99.6 | 99.5 | 99.6 | 99.6 | 99.8 | 100 | 100 |  | 99.9 | 99.8 | 99.8 | 99.7 | 98.8 | 99.2 | 99.5 | 99.5 | 99.3 | 99.8 | 99.5 | 99.5 | 88.7 | 73.8 |
| 11 | MW721926/WTV1/China | 99.4 | 99.3 | 99.6 | 99.5 | 99.6 | 99.6 | 99.8 | 100 | 100 | 100 |  | 99.7 | 99.8 | 99.7 | 98.8 | 99.3 | 99.5 | 99.5 | 99.3 | 99.7 | 99.5 | 99.4 | 88.7 | 73.7 |
| 12 | MW721925/WTV1/China | 99.4 | 99.3 | 99.6 | 99.5 | 99.6 | 99.6 | 99.8 | 100 | 100 | 100 | 100 |  | 99.8 | 99.9 | 98.9 | 99.1 | 99.5 | 99.5 | 99.3 | 99.9 | 99.6 | 99.6 | 88.6 | 73.7 |
| 13 | MW721924/WTV1/China | 99.4 | 99.3 | 99.6 | 99.5 | 99.6 | 99.6 | 99.8 | 100 | 100 | 100 | 100 | 100 |  | 99.9 | 99 | 99.1 | 99.6 | 99.6 | 99.4 | 99.9 | 99.7 | 99.7 | 88.6 | 73.8 |
| 14 | MW721922/WTV1/China | 99.4 | 99.3 | 99.6 | 99.5 | 99.6 | 99.6 | 99.8 | 100 | 100 | 100 | 100 | 100 | 100 |  | 98.9 | 99.1 | 99.6 | 99.6 | 99.4 | 99.9 | 99.6 | 99.7 | 88.6 | 73.8 |
| 15 | MZ965020/WTV1/China | 99.4 | 99.2 | 99.5 | 99.5 | 99.7 | 99.7 | 99.7 | 100 | 100 | 100 | 100 | 100 | 100 | 0.4 |  | 99.5 | 98.7 | 98.7 | 98.5 | 98.9 | 98.7 | 98.7 | 88 | 74.3 |
| 16 | MZ965019/WTV1/China | 99.4 | 99.3 | 99.6 | 99.5 | 99.6 | 99.6 | 99.8 | 100 | 100 | 100 | 100 | 100 | 100 | 0.2 | 100 |  | 98.8 | 98.8 | 98.6 | 99.1 | 98.8 | 98.8 | 88.3 | 74.4 |
| 17 | NC031304/WTV1/China | 99.4 | 99.3 | 99.6 | 99.5 | 99.6 | 99.6 | 99.8 | 100 | 100 | 100 | 100 | 100 | 100 | 0.1 | 100 | 100 |  | 100 | 99.5 | 99.6 | 99.4 | 99.3 | 88.8 | 73.5 |
| 18 | KM817660/WTV1/China | 99.4 | 99.3 | 99.6 | 99.5 | 99.6 | 99.6 | 99.8 | 100 | 100 | 100 | 100 | 100 | 100 | 0.1 | 100 | 100 | 100 |  | 99.5 | 99.6 | 99.4 | 99.3 | 88.8 | 73.5 |
| 19 | MW721921/WTV1/China | 99.4 | 99.2 | 99.5 | 99.5 | 99.6 | 99.6 | 99.7 | 100 | 99.9 | 100 | 100 | 100 | 100 | 0.3 | 99.9 | 100 | 100 | 100 |  | 99.4 | 99.2 | 99.1 | 88.6 | 73.4 |
| 20 | MW721923/WTV1/China | 99.4 | 99.2 | 99.5 | 99.5 | 99.6 | 99.6 | 99.7 | 100 | 99.9 | 100 | 100 | 100 | 100 | 0.1 | 99.9 | 100 | 100 | 100 | 99.9 |  | 99.7 | 99.7 | 88.6 | 73.7 |
| 21 | MW721929/WTV1/China | 99.4 | 99.2 | 99.5 | 99.5 | 99.6 | 99.6 | 99.7 | 100 | 99.9 | 100 | 100 | 100 | 100 | 0.3 | 99.9 | 100 | 100 | 100 | 99.9 | 99.9 |  | 99.4 | 88.6 | 73.7 |
| 22 | MW721928/WTV1/China | 99.4 | 99.1 | 99.4 | 99.4 | 99.6 | 99.6 | 99.6 | 99.9 | 99.8 | 99.9 | 99.9 | 99.9 | 99.9 | 0.3 | 99.8 | 99.9 | 99.9 | 99.9 | 99.8 | 99.8 | 99.8 |  | 88.5 | 73.8 |
| 23 | MN095536/WTV1/Thailand | 97 | 96.9 | 97.1 | 97 | 97.2 | 97.2 | 97.2 | 97.1 | 97.1 | 97.1 | 97.1 | 97.1 | 97.1 | 11.4 | 97.1 | 97.1 | 97.1 | 97.1 | 97 | 97 | 97 | 97 |  | 72.8 |
| 24 | MG764528/WTV1/Turkey | 95.7 | 95.5 | 96 | 95.9 | 96.1 | 96.1 | 96.1 | 95.9 | 96 | 95.9 | 95.9 | 95.9 | 95.9 | 22 | 96 | 95.9 | 95.9 | 95.9 | 95.9 | 95.9 | 95.9 | 95.8 | 95.7 |  |

Percent similarity at the complete nucleotide (upper triangle) and amino acid secquence of RdRp (lower triangle) levels calculated using ClustalW method implemented in MegAlign.

^*^WTV1, Wuhan tick virus 1.

**Supplementary Table S11. The sequence identity comparison of quaranjaviruses^*^**

|  |  | 1 | 2 | 3 | 4 | 5 | 6 | 7 | 8 | 9 | 10 | 11 | 12 | 13 | 14 | 15 | 16 | 17 | 18 | 19 | 20 |
| --- | --- | --- | --- | --- | --- | --- | --- | --- | --- | --- | --- | --- | --- | --- | --- | --- | --- | --- | --- | --- | --- |
| 1 | OM273645/GTQV/China |  | 78 | 58.8 | 60.9 | 60.7 | 60.5 | 71.8 | 53.7 | 54.2 | 54.3 | 53.2 | 52.4 | 51.2 | 49.8 | 51.6 | 50.9 | 51 | 49.8 | 48.8 | 51.4 |
| 2 | MH267793/Zambezi tick virus 1/Mozambique | 86.2 |  | 58.9 | 60.7 | 59.6 | 59.8 | 75.1 | 52.5 | 55.6 | 53.2 | 53.2 | 51.3 | 51.1 | 51.2 | 52.2 | 51.6 | 50.7 | 54.3 | 49.6 | 53 |
| 3 | NC025796/Wellfleet bay virus/USA | 54.3 | 51.7 |  | 71.8 | 71 | 71.6 | 59.7 | 54.7 | 55.6 | 55.2 | 54.8 | 51.4 | 51.9 | 51.3 | 53.1 | 50.8 | 51.5 | 50.8 | 48.3 | 50.5 |
| 4 | MG770333/Quaranfil quaranjavirus/India | 53.6 | 50.6 | 71.7 |  | 92.9 | 80 | 59.8 | 55.3 | 55 | 55.4 | 53.8 | 52.5 | 51.2 | 50.5 | 52.1 | 51.6 | 50.7 | 51.4 | 49.5 | 51.3 |
| 5 | NC038821/Quaranfil virus/Afghanistan | 53.7 | 50.6 | 71.3 | 97.6 |  | 79.1 | 59.2 | 55.4 | 55.3 | 55.5 | 53.6 | 52.4 | 51.4 | 50.2 | 51.5 | 51.1 | 50.7 | 50.8 | 49.1 | 50.9 |
| 6 | NC052931/Johnston atoll quaranjavirus/New Zealand | 54.1 | 51 | 71 | 84.8 | 83.9 |  | 58.9 | 55.3 | 55.4 | 55.2 | 55 | 52.9 | 51.4 | 51.2 | 52.3 | 51.8 | 50.4 | 51.2 | 49.6 | 51.9 |
| 7 | MZ502305/Granville quaranjavirus/Trinidad and Tobago | 72.1 | 75.3 | 50.1 | 48.8 | 49 | 49 |  | 52.9 | 55.2 | 53.1 | 53.3 | 51.8 | 52.3 | 52.1 | 53.1 | 50.6 | 53 | 53.1 | 48.7 | 55 |
| 8 | KM817616/Jiujie fly virus/China | 33.2 | 35.8 | 35.4 | 34.9 | 34.7 | 34.8 | 41.3 |  | 66.5 | 63.1 | 63.5 | 60.9 | 49.2 | 50.9 | 50.2 | 48.2 | 48 | 50.3 | 47.6 | 50.5 |
| 9 | KM817618/Shayang spider virus 3/China | 24.6 | 26.8 | 27.7 | 25 | 24.8 | 25.5 | 25.5 | 27.9 |  | 62.5 | 64.7 | 60.3 | 49.8 | 51.9 | 52.7 | 49.8 | 50.3 | 50.9 | 48.8 | 50.6 |
| 10 | MH558140/Longchuang virus/China | 37.4 | 36.6 | 38.7 | 38.6 | 38 | 37.8 | 34 | 41.1 | 42.3 |  | 65.2 | 62.1 | 50.2 | 50.3 | 51.5 | 48.8 | 48.4 | 48.5 | 47.9 | 49.5 |
| 11 | MW033638/Soybean thrips quaranja-like virus 2/USA | 40.6 | 38.7 | 40.9 | 40.9 | 40.4 | 40.9 | 37 | 47.1 | 33.5 | 55.5 |  | 63.2 | 50.2 | 50.2 | 51.9 | 49.9 | 49.8 | 47.7 | 49.7 | 50.8 |
| 12 | MW256691/Bemisia tabaci quaranja-like virus/Israel | 37.5 | 39.7 | 38.1 | 38.4 | 38.1 | 39 | 40.7 | 49 | 32.3 | 47.9 | 54.7 |  | 48.2 | 48 | 48.3 | 46 | 47.6 | 49.3 | 46.5 | 48 |
| 13 | KM817619/Shuangao insect virus 4/China | 38.3 | 38 | 39 | 38.3 | 38 | 37.8 | 36 | 28.5 | 21.5 | 33.4 | 34 | 32.8 |  | 65.2 | 66.3 | 61 | 62.4 | 52.6 | 53.6 | 53.1 |
| 14 | KM817620/Wuhan louse fly virus 3/China | 35.9 | 35.5 | 34.7 | 35.9 | 35.5 | 35.1 | 34.6 | 26.4 | 18.4 | 30.3 | 32.9 | 31.3 | 52.1 |  | 69 | 61.6 | 62.6 | 52.1 | 52.1 | 53 |
| 15 | KX883875/Jingshan fly virus 1/China | 38.3 | 38.6 | 37.9 | 37.5 | 36.8 | 38.1 | 37 | 28.1 | 22.3 | 33.8 | 34.9 | 32.7 | 62.8 | 52.8 |  | 62 | 64 | 50.4 | 55.1 | 52.6 |
| 16 | MW434253/Guadeloupe mosquito quaranja-like virus 1/USA | 36.7 | 36.5 | 35.5 | 35.5 | 35.2 | 36.6 | 35.2 | 28.6 | 21 | 32.7 | 34.5 | 31.4 | 52.9 | 52.7 | 53 |  | 66.4 | 50.1 | 53.9 | 51.6 |
| 17 | MW452276/Wuhan mosquito virus 5/China | 37.1 | 36.6 | 37 | 35.7 | 35.1 | 36.1 | 35.7 | 27.7 | 22.6 | 32.1 | 34.2 | 32.2 | 55.6 | 56.2 | 55.1 | 61.5 |  | 51.2 | 55.1 | 52.1 |
| 18 | KM817627/Wuhan mothfly virus/China | 35.9 | 39 | 35.7 | 36.5 | 35.9 | 36.1 | 43.8 | 43.1 | 21.5 | 30.6 | 31.4 | 37.1 | 40.3 | 36.7 | 36.8 | 37.1 | 38.3 |  | 47 | 50.8 |
| 19 | MK440648/Wuhan mosquito virus 6/Sweden | 33.4 | 32.9 | 31.9 | 32.8 | 32.1 | 33.3 | 31.6 | 25.6 | 20.5 | 30.9 | 31.5 | 28.1 | 43 | 42.4 | 44 | 42.9 | 43 | 31.3 |  | 49.6 |
| 20 | KM817626/Wuhan mosquito virus 7/China | 37.2 | 39.7 | 34 | 34.8 | 34.6 | 34.6 | 39.2 | 32.8 | 24.7 | 32.1 | 33.1 | 34 | 38.5 | 37.2 | 37.3 | 36 | 37.3 | 38.7 | 35.7 |  |

Percent similarity at the complete nucleotide (upper triangle) and amino acid secquence of PB1 (lower triangle) levels calculated using ClustalW method implemented in MegAlign.

^*^GTQV, Guangdong tick quaranjavirus
